# Condensates formed by prion-like low-complexity domains have small-world network structures and interfaces defined by expanded conformations

**DOI:** 10.1038/s41467-022-35370-7

**Published:** 2022-12-13

**Authors:** Mina Farag, Samuel R. Cohen, Wade M. Borcherds, Anne Bremer, Tanja Mittag, Rohit V. Pappu

**Affiliations:** 1 Department of Biomedical Engineering and Center for Biomolecular Condensates, Washington University in St. Louis, St. Louis, MO USA; 2 Department of Structural Biology, St. Jude Children’s Research Hospital, Memphis, TN USA

**Keywords:** Computational biophysics, Biophysical chemistry

## Abstract

Biomolecular condensates form via coupled associative and segregative phase transitions of multivalent associative macromolecules. Phase separation coupled to percolation is one example of such transitions. Here, we characterize molecular and mesoscale structural descriptions of condensates formed by intrinsically disordered prion-like low complexity domains (PLCDs). These systems conform to sticker-and-spacers architectures. Stickers are cohesive motifs that drive associative interactions through reversible crosslinking and spacers affect the cooperativity of crosslinking and overall macromolecular solubility. Our computations reproduce experimentally measured sequence-specific phase behaviors of PLCDs. Within simulated condensates, networks of reversible inter-sticker crosslinks organize PLCDs into small-world topologies. The overall dimensions of PLCDs vary with spatial location, being most expanded at and preferring to be oriented perpendicular to the interface. Our results demonstrate that even simple condensates with one type of macromolecule feature inhomogeneous spatial organizations of molecules and interfacial features that likely prime them for biochemical activity.

## Abstract (web summary)

The authors provide a physical description for condensates as complex fluids defined by small-world network structures for their interiors, and of interfaces featuring a preference of locally and globally expanded conformations.

## Introduction

In living cells, many proteins and nucleic acids are concentrated into membraneless biomolecular condensates that form and disassemble at the right place and time^1–4^. Coupled associative and segregative phase transitions (COAST) of multivalent associative macromolecules (MAMs) help explain how condensates form and dissolve in response to environmental, mechanical, chemical, and developmental cues^5,6^. Phase separation coupled to percolation^5,6^, complex coacervation^7^, and polymerization-induced phase separation^8^ are examples of COAST-like processes involving MAMs. Multivalence of domains or motifs that form reversible physical crosslinks are defining features of proteins that are biologically relevant drivers of condensate formation^1,4,5^. Recent attention has focused on intrinsically disordered protein domains known as prion-like low complexity domains (PLCDs)^9^. These domains are present in and drive functional as well as aberrant phase transitions of many biomolecular condensates such as cytosolic stress granules and processing bodies^10,11^. PLCDs are also found tethered to a range of different RNA and DNA binding domains that drive the formation of distinct types of cytosolic and nuclear condensates^12^.

The compositional makeup of PLCD sequences is distinctive. Roughly 60-70% are polar residues, 15–20% of the residues are aromatic π-systems, and the remainder are ionizable residues^13^. Within each PLCD, the aromatic residues are distributed uniformly across the linear sequence^9,14,15^. Although sequences of PLCDs vary considerably across evolution, the compositional biases and linear patterning of aromatic residues are conserved features^13,14^. Recent experimental work has uncovered the physiochemical principles underlying the connections between sequence-encoded features and the driving forces for COAST-like phase transitions of PLCDs. These studies used the PLCD from the protein hnRNPA1, hereafter referred to as the A1-LCD or wild-type (WT) A1-LCD, and designed variants thereof as targets for investigation^9,13^.

PLCDs are biological instantiations of linear associative polymers^5,16–24^. Such systems are defined by sticker-and-spacer architectures, wherein the driving forces for phase separation are governed by the interplay between chemistry-specific physical crosslinks among stickers, and the effective, solvent-mediated interactions among spacers^5,23,25^. Accordingly, the phase behaviors of sticker-and-spacer systems are characterized as phase separation coupled to percolation (PSCP)^4,5,23–26^. This COAST-like process generates condensates that are microgel-like^27,28^, implying that the physically crosslinked networks of molecules are condensate spanning^5,23,25^. Such systems will be viscoelastic in nature and their material properties should be governed by emergent structures of the underlying networks, including the conformations of individual molecules, the extent of crosslinking they enable, the topological structures generated by crosslinking, and the impacts of spacers on the dynamics of intermolecular rearrangements that drive the making and breaking of crosslinks^6,29,30^.

In this work, we go beyond mean-field models that were used in recent studies^13^. We investigate the molecular and mesoscale organization of PLCDs within, outside, and at the interfaces of condensates. We leverage residue-level descriptions afforded by simulations that use LaSSI, a bond fluctuation-based lattice model paradigm^23^, that we adapt to reproduce the macroscopic phase behavior of the A1-LCD system and numerous designed variants of this system^13^. Unlike mean-field models, the LaSSI engine allows us to account for chain connectivity, excluded volume effects, and site-specific interactions. The totality of all these effects on PSCP of PLCDs is evaluated. Our analysis of structural properties of condensate interiors, interfaces, and coexisting dilute phases yields insights into complexities that are manifest even for condensates formed by seemingly simple systems such as PLCDs with sticker-and-spacer architectures.

## Results

### Computational sticker-and-spacer model for A1-LCD and designed variants that is transferable unto PLCDs in general

Coarse-grained models are often deployed to describe the sequence-specific phase transitions of intrinsically disordered proteins. To understand how context-dependent specificity enables phase transitions for a specific class of sequences, we use either a parsimonious set of experimental data or fine-grained, system-specific simulations to learn a model that can apply to the sequences used in the parameterization. The parameters are transferable to sequences that share similar compositional or architectural biases. In our case, this includes the family of PLCDs. The parameterization uses the Gaussian process Bayesian optimization, described previously^31^. We adapted this approach for developing a model to be deployed in lattice-based Monte Carlo simulations that use contact-based potentials. Specifically, we used LaSSI, which is a lattice-based simulation engine for coarse-grained simulations of sequence- and / or architecture-specific PSCP of biopolymers. The development of LaSSI was inspired by the bond fluctuation model for lattice polymers^32,33^, and a generalization developed by Shaffer^34^. In the current implementation, we adapted LaSSI for modeling PLCDs using a single bead-per-residue. There are nine specific residue types, one each for tyrosine (Y), phenylalanine (F), arginine (R), lysine (K), glycine (G), serine (S), threonine (T), glutamine (Q), asparagine (N), and a generic residue (X). The contact energies between pairs of sites occupied by the different residue types were parameterized using a protocol described in the Supplementary Methods and summarized in Supplementary Fig. 1.

**Fig. 1:**
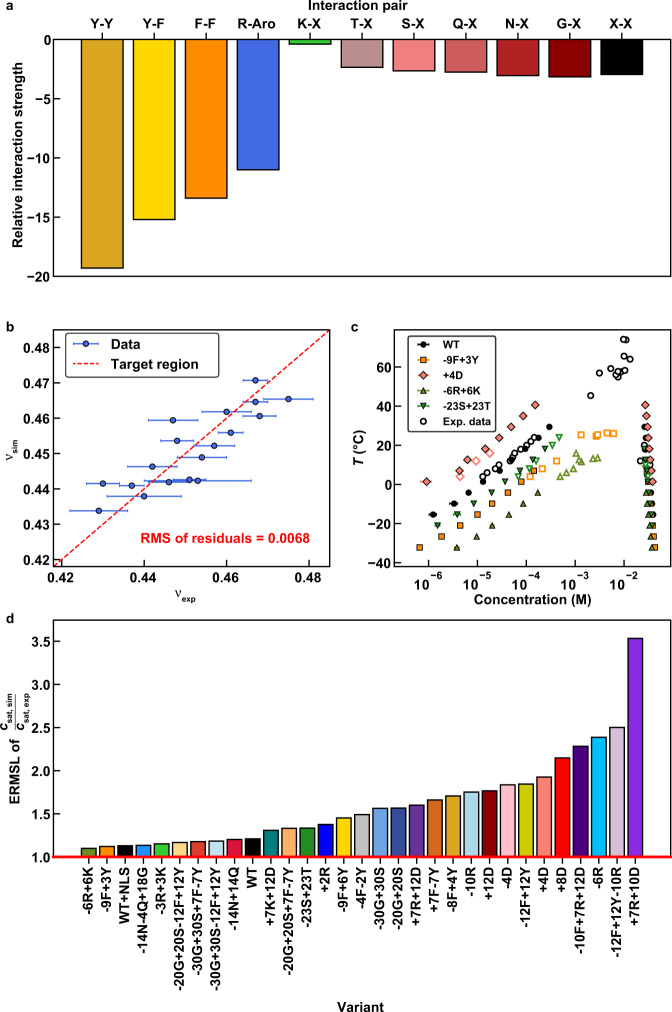
Setup and assessment of the computational model. **a** Pairwise interaction strengths used in the computational model. Amino acids are referred to by single-letter codes. “X” is used to indicate any amino acid for which a specific interaction is not defined. “Aro” is used to indicate either tyrosine or phenylalanine. Contact energies for Y-Y, Y-F, F-F, R-Aro, K-X, and X-X were parameterized using Gaussian process Bayesian optimization (GPBO; see supplemental material and Supplementary Fig. 1). All other energies were parameterized by matching experimental and computational phase diagrams of “spacer” variants^13^ (see Supplementary Methods). **b** *R*_*g*_ values scale with chain length according to the relation *R*_*g*_ ~ *N*^ν^. Here, ν is actually an apparent scaling exponent ν_app_, that is sequence specific, and is extracted from SEC-SAXS data using the approach developed by Riback et al.^79^. We compare values of ν obtained by fitting SEC-SAXS data to a molecular form factor (ν_exp_) to those obtained from single-chain LaSSI simulations (ν_sim_) and use GPBO to parameterize a computational model. Each data point corresponds to a unique A1-LCD variant. The red dashed line represents the regime where ν_exp_ = ν_sim_, and the root mean squared error is calculated using the residuals from this line. Vertical error bars representing the standard error about the mean across five replicates are smaller than the markers. Horizontal error bars represent the uncertainty from fitting the SAXS data to molecular form factors. **c** Calculated coexistence curves or binodals (solid markers) of various A1-LCD variants plotted alongside experimentally derived binodals (open markers). Temperature and concentration are converted from simulation units to Kelvin and molar units, respectively, using the scaling factors of Martin et al.^9^. Error bars represent standard errors from the mean across 3 replicates. **d** ERMSL (see Supplementary Methods) comparing experimentally measured (*c*_sat,exp_) and computationally derived (*c*_sat,sim_) saturation concentrations. Source data are provided as a Source Data file.

Size exclusion chromatography-aided small-angle x-ray scattering (SEC-SAXS) data were collected for the A1-LCD and a set of designed variants^13^. These data provide an estimate of the ensemble-averaged radius of gyration (*R*_*g*_) for each of the PLCDs at 25 °C in the one-phase regime, where care is taken to ensure that proteins do not undergo phase separation or oligomerization^13^. We then developed a model for the contact energies among all unique pairs of residue types using the following protocol: We performed simulations of individual chains, computed the correlation between LaSSI-derived and measured chain dimensions, and iterated to convergence via a Gaussian process Bayesian optimization approach developed in previous work^31^. Details are furnished in the Methods section and Supplementary Methods. The resultant model for the contact energies is summarized in Fig. 1a.

We use Metropolis Monte Carlo simulations to sample configurational space for single chains and multiple chains on a cubic lattice^23^. Accordingly, the transition probability for converting between pairs of configurations is proportional to exp(-∆*E*/*k*_*B*_*T*). Here, ∆*E* is the difference in energy between a pair of configurations. In the simulations, we set *k*_B_ = 1, and *T* is in the interval 40 ≤ *T* ≤ 60. In units of the dimensionless simulation temperature, replacing Y-Y interactions with a Y-K interaction, which represents the largest change in ∆*E*, will range from ≈ 0.32 *k*_*B*_*T* to 0.47 *k*_*B*_*T*, depending on the simulation temperature. That the model reproduces the target function against which it was parameterized is evident in Fig. 1b, which shows a strong positive correlation between the apparent scaling exponents inferred from SEC-SAXS measurements and from the LaSSI simulations of individual chain molecules.

Note that our parameterization of the model rests on the assumption of strong coupling between the driving forces for single-chain compaction and phase separation^9,35,36^. Bremer et al., showed that this coupling breaks down for variants where the net charge per residue (NCPR) deviates from zero in a way that does not impact single-chain dimensions, but does impact multi-chain interactions^13^. Based on the analysis of Bremer et al.^13^, we included a mean-field NCPR-based adjustment to the potentials for simulations of multichain phase behavior. In these simulations, the pairwise interactions were weakened or strengthened by an amount that is proportional to the difference in NCPR values between that of the given variant and that of the wild type A1-LCD (see Supplementary Methods for details).

### Judging the accuracy of computed binodals

We computed two-phase coexistence curves (binodals) for 31 different sequences, including the wild-type A1-LCD (Supplementary Fig. 2). Results for the wild-type and four of the variants studied by Bremer et al.^13^, are shown in Fig. 1c. The computed and measured binodals show good agreement with one another. Further, for each of the 31 sequences, we calculated the exponential root mean square log (ERMSL) between the measured and computed low-concentration arms of binodals (see Methods). The ERMSL is a positive value greater than or equal to one. An ERMSL value of ten indicates that, on average, the concentrations along the low-concentration arm of a binodal differ by order of magnitude from the measured values. Alternatively, an ERMSL value of 1 indicates that there is no error between the dilute arms and that they overlay perfectly. For all but one of the sequences, the ERMSL is ≤2.5 (Fig. 1d). This shows that the model reproduces measured phase boundaries, specifically the low concentration arms of the binodals, for all experimentally characterized variants even though we parameterized the model using SEC-SAXS data for only 50% of the sequences.

### Testing the transferability of our model

Given the accuracy of our model in recapitulating the measured binodals of A1-LCD and variants thereof, we asked if it could accurately represent other PLCDs. To test for transferability, we measured the coexistence curve of the PLCD from the protein Fused in Sarcoma (FUS). The computed and measured binodals were compared, as shown in Supplementary Fig. 3, yielding an ERMSL of ~2.7 for the low-concentration arm of the binodal. This represents good agreement between experiments and simulations even though the parameterization of the model did not use any data for the FUS-LCD. We attribute this transferability to the fact that both sequences are PLCDs that share similar non-random patterns of residues with respect to one another along the linear sequence. To make this point, we performed an analysis using the recently introduced NARDINI algorithm^14^ to quantify the extent to which the A1-LCD and FUS-LCD systems resemble one another, not just in compositional biases, but also in terms of non-random sequence patterns (Supplementary Fig. 4). We find that A1-LCD and the FUS-LCD share similar non-random binary patterns, such as the uniform distribution of aromatic residues, the presence of blocks of glycine residues, and segregative patterning between glycine residues and polar residues (Ser, Thr, Asn, Gln, Cys, His). These features have recently been shown to be preserved across a wide-range of PLCDs^14^. Next, we analyzed the sequence of the intrinsically disordered region (IDR) of DDX4 protein, which is classified as an RGG domain. This IDR has a compositional bias that is shared with PLCDs. However, the NARDINI-based analysis shows that the non-random binary patterns in the DDX4-IDR are very different from those of PLCDs (Supplementary Fig. 4). As a result, the current LaSSI model, designed for PLCDs, is unlikely to capture the phase behavior of RGG domains. This is because a fully transferable model must account for built-in context-dependencies, and this requires the inclusion of three-body terms that modulate two-body interactions^24^. These have not yet been incorporated into the interaction models for LaSSI or any other simulation paradigms. Having established the validity of the computational model for describing the phase behaviors of PLCDs, we turn our attention to connecting the molecular and mesoscale properties of condensates obtained from LaSSI-based simulations.

### Conformations in dense phases are more expanded compared to the coexisting dilute phases

**Fig. 2:**
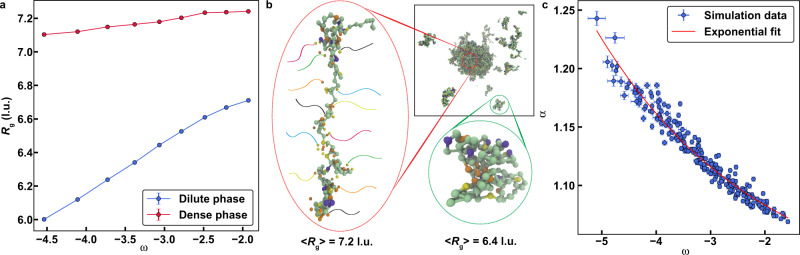
Comparison of conformations within dense vs. dilute phases. **a** *R*_*g*_ of chains in the dilute (blue) and dense phase (red) derived from condensates of the wild-type A1-LCD plotted against the width of the two-phase regime (see text). **b** A schematic depicting how intramolecular sticker-sticker interactions promote chain compaction in the dilute phase, whereas distinct intermolecular sticker-sticker interactions promote chain expansion in the dense phase. Here, each chain is depicted as a flexible tail of distinct color. **c** Swelling ratio, which quantifies the degree of expansion of chains in the dense phase relative to the dilute phase, is plotted against the width of the two-phase regime for all A1-LCD variants in this study. The datapoints collapse onto a single exponential curve (solid red curve; see Methods for fitting model and parameters). Error bars represent standard errors about the mean across 3 replicates. l.u. is lattice units. Source data are provided as a Source Data file.

We quantified the *R*_*g*_ values of individual chain molecules in coexisting dilute and dense phases. The results are shown in Fig. 2a for the wild-type A1-LCD. Here, *R*_*g*_ is plotted against the parameter $\omega (T)={{{{\rm{log }}}}}_{10}\left[\frac{{c}_{{{{{{\rm{dilute}}}}}}}(T)}{{c}_{{{{{{\rm{dense}}}}}}}(T)}\right]$, which is the temperature-dependent width of the two-phase regime (Supplementary Fig. 5a). Note that ω is negative because the concentration in the dilute phase (*c*_dilute_) is lower than the concentration in the dense phase (*c*_dense_). Also note that ω increases with *T* and approaches zero as *T* approaches the critical temperature *T*_*c*_ ≈ 49 °C beyond which the system is in the one-phase regime. The conversion between simulation temperatures and degree-Celsius is based on the approach of Martin et al.^9^. In both the dilute and dense phases, the *R*_*g*_ values of individual molecules increase as *T* increases (Fig. 2a). However, for each of the temperatures that are below *T*_*c*_, the *R*_*g*_ values in the dense phase are systematically higher than *R*_*g*_ values in the dilute phase (Fig. 2a). This is due to the network of intermolecular interactions that are realized in the dense phase as opposed to the intramolecular interactions in the dilute phase – a feature that is shown pictorially in Fig. 2b where distinct intermolecular interactions are depicted as tails of different colors emanating from sticker residues.

Recently, Hazra and Levy showed that generic polymers featuring a mixture of long- and short-range interactions are more expanded in dense vs. coexisting dilute phases^37^. Given observations of similar phenomena using very different models^37,38^, we analyzed results for variants where we either titrated the number of aromatic stickers or we altered the identities of the aromatic stickers Y vs. F. The goal was to assess the robustness of chain swelling across the phase boundary.

We computed the swelling ratio α, defined as the ratio of *R*_*g*_ in the dense phase to *R*_*g*_ in the dilute phase. We note that α approaches unity as *T* approaches *T*_c_ (Supplementary Fig. 5b). As with A1-LCD, we find that the mutational variants are more expanded in the dense phase when compared to the dilute phase (Fig. 2c). In a plot of α against ω (Fig. 2c), we find that the swelling ratios for all A1-LCD variants collapse onto a single master curve without any adjustable parameters. This curve can be fit to an exponential decay function (Fig. 2c). It implies that knowledge of the width of the two-phase regime for a disordered PLCD should allow us to infer the swelling ratio from the master curve. Further, if we supplement knowledge regarding the width of the two-phase regime with measurements of chain dimensions in the dilute phase, then we can use a master curve to infer the average *R*_*g*_ values of individual chain molecules in the dense phase.

The exponential decay function for the swelling ratio implies that the solvent qualities of the dense and dilute phases approach each other continuously. This deviates from the crossover behavior that is expected from Landau theory^39^ and demonstrated for long homopolymers^40^ such as polystyrene in methylcyclohexane. Crossover theories predict that in three-dimensions, the width of the two-phase regime scales as (*T*_*c*_ *– T*)^0.33^ in the vicinity of the critical temperature *T*_*c*_. However, away from *T*_*c*_, the width scales as (*T*_*c*_ *– T*)^0.5^. The existence of this crossover behavior places infinitely long homopolymers in the same universality class as the 3D Ising model. Our observations, details of which are discussed in the Supplementary Discussion, suggest a different behavior with continuous decay, implying the lack of a crossover between mean-field and critical regimes. This might be because the critical regime for finite-sized heteropolymers is vanishingly small or because large-scale fluctuations are present across the entire two-phase regime. The apparent concordance with the master curve shown in Fig. 2c invites further investigations into how the critical regime must be described for finite-sized, heteropolymeric sticker-and-spacer systems. This requires the development of models for a suite of disordered proteins and studying how the width of the two-phase regime changes as *T* approaches *T*_*c*_.

Next, we analyzed the physical basis for conformational differences across the phase boundary by assessing the three-way interplay of intra-chain, inter-chain, and chain-solvent contacts as determinants of *R*_*g*_ in the dense phase (Supplementary Fig. 6). Here, chain-solvent contacts refer to the observation of a vacant site adjacent to a site occupied by a chain. Our analysis shows that the sole determinant of the extent of chain compaction is the fraction of intramolecular contacts (*f*_intra_) (Supplementary Fig. 6). For a given *R*_g_ value, which fixes *f*_intra_, the sum of the fractions of inter-chain (*f*_inter_) and chain-solvent contacts (*f*_sol_) is constrained: *f*_intra_ + *f*_inter_ + *f*_sol_ = 1. Accordingly, *f*_inter_ + *f*_sol_ = (1 – *f*_intra_), and hence any increase in *f*_sol_ is compensated by a decrease in *f*_inter_ and vice versa (Supplementary Fig. 6).

### Networking of chains within dense phases is determined by the strengths and valence of stickers

From the contact energies (ε) summarized in Fig. 1a we note that the magnitudes of interaction energies of stickers follow a hierarchy whereby ε_YY_ > ε_YF_ > ε_FF_ > ε_RY/F_. Therefore, it follows that tyrosine (Y), and phenylalanine (F) are the primary stickers whereas arginine (R) is an auxiliary sticker in PLCDs. This hierarchy is likely to be distinct for distinct sequence families.

Stickers form reversible crosslinks, and in the lattice simulations a crosslink is distinguished from a random contact by the frequency of observing a specific pair of residues coming into contact. Crosslinking is governed by the hierarchy of interaction energies and the temperature. We quantified a ratio of association *g*_*a*_, which we define as ${g}_{a}=\frac{{p}_{a,{{{{{\rm{seq}}}}}}}}{{p}_{a,{{{{{\rm{ref}}}}}}}}$. Here, *p*_*a*,seq_ is the relative probability of observing sticker-sticker vs. sticker-spacer contacts in the sequence (seq) of interest. The parameter *p*_*a*,ref_ is the homopolymer equivalent of *p*_*a*,seq_. The homopolymer is of the same length as the wild-type A1-LCD. The contact energies, which are identical among all residues, are parameterized to reproduce the computed binodals for the wild-type A1-LCD. For comparative analysis, we impose the sticker-and-spacer architecture of the wild-type sequence onto the homopolymer (Supplementary Fig. 7).

The ratios of association were computed for different sequence variants of the A1-LCD system (Supplementary Fig. 8a–c). Replacing all phenylalanine residues with tyrosine increases the ratio of association (see data for -12F+12Y in Supplementary Fig. 8a), whereas replacing all tyrosine residues with phenylalanine lowers the ratio of association (see data for +7F-7Y in Supplementary Fig. 8a). Decreasing the valence of aromatic residues, whereby six of the stickers in A1-LCD are replaced by spacers, lowers the ratio of association to be below one. This implies that the extent of networking is weakened even when compared to the equivalent homopolymer (see data for -4F-2Y in Supplementary Fig. 8a).

Surprisingly, replacing auxiliary stickers such as arginine with a spacer that weakens the driving forces for phase separation increases the ratios of association when compared to the wild-type A1-LCD (see data for -3R+3K and -6R+6K compared to the wild-type A1-LCD in Supplementary Fig. 8a; also Supplementary Fig. 2e). This is because the auxiliary stickers compete with the primary aromatic stickers. However, even though the ratio of association of stickers is higher in variants with fewer arginine residues, the driving forces for phase separation are weakened by the competing effects of spacers with a higher preference to be solvated. These observations point to the competing and separable effects of specific interactions vs. spacer-mediated solubility – a feature that has been argued to be unavailable for intrinsically disordered proteins^41^ but is clearly demonstrated to be prevalent in our analysis.

In general, changes to the identities and hence interactions mediated by spacers have a negligible effect on the ratios of association as shown in our results for thirteen different variants where the identities and hence interactions mediated by spacers have been altered substantially (Supplementary Fig. 8b, c). When compared to data for measured and computed binodals (see Supplementary Fig. 2), we conclude that solubility-determining interactions involving spacers can impact the driving forces for phase separation without affecting the networking of stickers. Taken together, these results demonstrate that specific sequence features may affect driving forces for phase separation and internal condensate organization in non-equivalent ways. From a protein engineering standpoint, this aspect of the sequence encoding could enable the design and identification of separation of function mutations^6^.

Next, we quantified the probability *P*(*s*) of realizing clusters of lattice sites within condensates with *s* stickers that form via inter-sticker crosslinks. Although the distributions (shown in Supplementary Fig. 8d for the wild-type A1-LCD) are exponentially bounded for small *s*, they have heavy tails. This feature also appears in the probability density for self-avoiding walks^42^, with the difference being that the heavy tails in our case are created by the crosslinking of stickers. We fit the data for *P*(*s*) to the functional form for the cumulative distribution function of a discrete Weibull distribution^43^ given by:

1 $$P(s)=1-\exp \left[-{\left(\frac{s+1}{\lambda }\right)}^{k}\right].$$

Here, *s* is the number of stickers within each cluster, whereas λ and *k* are, respectively, sequence-specific scale and shape parameters of the Weibull distribution. The sequence-specific values of λ and *k* were extracted by linear regression analysis of plots of ln[–ln(1–*P*(*s*))] vs. ln(*s* + 1). As shown in Supplementary Fig. 8e, increasing the strength of stickers (-12F+12Y) leads to increased clustering of stickers (larger λ-values) when compared to wild-type A1-LCD. Likewise, decreasing the strengths of stickers (+7F-7Y) lowers the extent of clustering of stickers (lower λ-values) when compared to the wild-type A1-LCD. Lowering the valence of stickers significantly lowers the extent of clustering (see data for -4F-2Y in Supplementary Fig. 8e). Finally, we note that the extent to which large clusters of stickers are formed, quantified by the values of *k*, where lower values imply heavier tails, is governed almost exclusively by the valence of stickers (Supplementary Fig. 8f).

### Condensates form small-world structures defined by networks of physical crosslinks

**Fig. 3:**
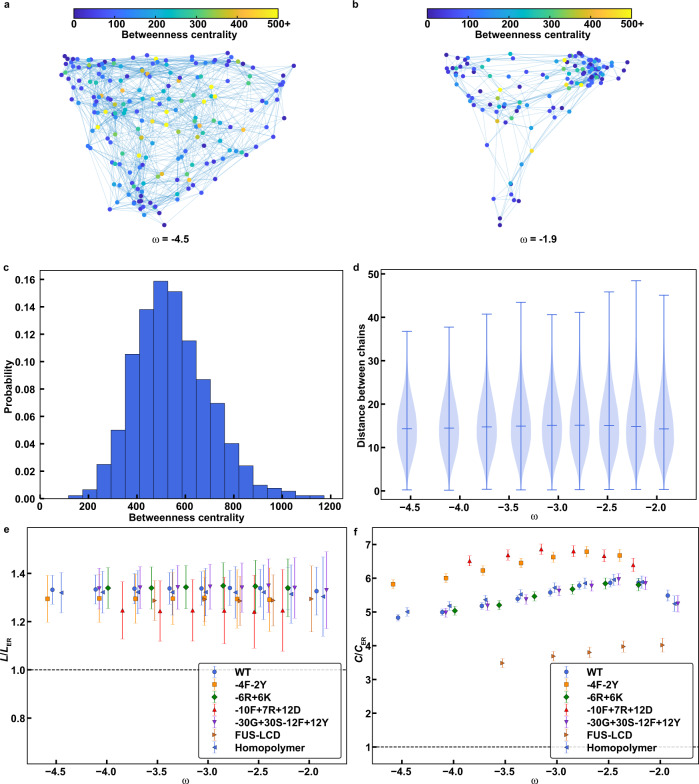
The interiors of condensates form small-world network structures. **a**, **b** Representative graphs for the largest connected cluster at the largest **(a)** and smallest **(b)** absolute value of ω (as defined in Fig. 2). As ω approaches zero, the system approaches the critical point. Results are shown here from analyses of simulations for the wild-type A1-LCD. The nodes represent individual molecules and are colored according to their betweenness centralities as defined in Eq. (2). Two chains are connected by an undirected edge if any two stickers between them are within $\sqrt{3}$units on the cubic lattice. **c** The betweenness centrality distribution for chains with degree 24 at the lowest value of ω, −4.5, for the WT A1-LCD. 920 samples were used to generate the distribution. **d** The pairwise distance distributions of the 5% of chains with the highest betweenness centralities at various ω values for the WT A1-LCD. In each violin plot, the tails extend to the minimum and maximum values, and the median is annotated. Each violin plot is created using 1.35 × 10^5^ data points. **e**, **f** the average path length, *L* **(e)**, and the average clustering coefficient, *C* **(f)**, for a diverse set of 7 constructs, including the WT A1-LCD. The values shown here are normalized by the corresponding Erdős-Rényi values for random graphs. The dashed horizontal line represents the values that would be expected assuming an Erdős-Rényi model^45^. The error bars represent the standard deviation about the mean across 10 replicates for the WT, 5 replicates for the FUS-LCD, and 3 replicates for all other sequences. Source data are provided as a Source Data file.

The heavy-tailed nature of the cluster distributions suggests that molecules can be networked to be condensate spanning. This would generate specific types of network structures, which we analyzed using graph-theoretic methods^44^. In this analysis of the simulation results, we treat each molecule within a condensate as a node. An undirected edge is drawn between a pair of nodes if at least one pair of stickers from the molecules in question forms a contact. The resultant graphs depicting the representative topological structures at a given snapshot are shown for the WT A1-LCD at the highest and lowest temperatures (Fig. 3a, b). Each node is colored by its *betweenness centrality*, a measure of connectedness that is defined as:

2 $$g(n)=\mathop{\sum}\limits_{s\ne n\ne t}\frac{{\sigma }_{st}(n)}{{\sigma }_{st}}$$

Here, *g*(*n*) is the betweenness of a given node, σ_*st*_ refers to the total number of shortest paths (defined by the fewest number of connected nodes) from node *s* to node *t*, and σ_*st*_(*n*) is the number of those paths that go through *n*.

We plot the betweenness centrality distribution of the WT A1-LCD at the lowest temperature, indicated by ω, for chains with a degree equal to 24 (Fig. 3c). Here, the degree is defined as the total number of distinct chains with which the chain in question interacts. We choose a single, large degree, in this case 24, to disentangle the positive correlation between degree and betweenness (Supplementary Fig. 9a). The distribution is skewed to the right, indicating a population of central nodes, or hubs, with both large betweenness and large degree. We repeated this analysis with other large degree values, and consistently found a right-skewed distribution, a behavior that suggests the presence of exceptionally well-connected hubs (Supplementary Fig. 10).

We also calculated the distribution of distances between the most central chains in the condensate, defined as those with the top 5% betweenness centralities (Fig. 3d). At all values of ω, the distance distribution appears broad, with a median of ~15 lattice units. In Supplementary Fig. 11 (discussed below), we show that the radius of the dense phase, excluding the interface, is ~20–30 lattice units. Thus, the distance distributions suggest that the most central chains are not clumped together in a single region within the condensate, but rather are distributed throughout the condensate. We repeated the analysis in Fig. 3d for different polymers, including -4F-2Y, -6R+6K, -10F+7R+12D, -30G+30S-12F+12Y, the FUS-LCD, and the homopolymer equivalent of WT A1-LCD. These constructs were chosen to represent diverse sequence features, including varied sticker valences / strengths, spacer solvation preferences, and polymer lengths. We plotted the average distances between the most well-connected chains at various ω values and found that, in general, the average distance increases as ω approaches 0 (Supplementary Fig. 9b). There is also a correlation with sticker valence, whereby chains with lower sticker valences than WT A1-LCD such as -4F-2Y and -10F+7R+12D show higher average distances. Condensates comprising these variants have a similar number of chains (nodes) as those comprising the WT A1-LCD, but fewer sticker-sticker interactions (edges). This result suggests that as the total number of sticker-sticker interactions decreases, without changing the total number of chains in the condensate, the most central chains are more likely to be distributed throughout the condensate.

Finally, we performed simulations of the WT A1-LCD involving only local Monte Carlo moves (see Supplementary Methods for details) to understand how the condensate structure varies over time. For these analyses, we consider the number of Monte Carlo moves as a proxy for time. In this analysis, the shortest “timescale” we use is significantly larger than the average sticker-sticker lifetime (Supplementary Fig. 12). First, we calculated the root-mean-square-displacement of chains, binned by their betweenness centralities (Supplementary Fig. 13a). We find that non-central chains typically move over larger distances than more central chains after a given number of Monte Carlo moves. Therefore, centrality hinders molecular transport.

We also calculated the probability that a chain whose betweenness centrality is in the top 5% stayed in the top 5% after a given number of Monte Carlo moves (Supplementary Fig. 13b). Assuming completely uncorrelated networks, this percent probability would be exactly 5%. In contrast, we calculate a percent probability anywhere from 15% to 35% depending on the simulation temperature, and the number of Monte Carlo moves. Relating this with our analysis of root mean squared displacement (RMSD) values (Supplementary Fig. 13a), we find that larger numbers of steps (akin to longer times) result in RMSD values that are approximately three times greater than those associated with shorter timescales. However, the likelihood that a chain stays well-connected only decreases by ~30% (Supplementary Fig. 13b), suggesting that even as chains move through the condensate, there is a persistent memory of the network structure.

Taken together, the results presented above, namely, the right-skewed distributions of betweenness centrality, the observation that well-connected chains are distributed throughout the condensate, and the relationship between chain connectedness and mobility, suggest a small-world structure of percolated networks within condensates, wherein a small subset of chains in the condensate behave as highly interconnected hubs. To test this hypothesis, we computed two standard measures of graph topology, the relative path lengths and relative clustering coefficients of condensate graphs, at different temperatures, by referencing these parameters to values obtained from Erdős-Rényi random graphs^45^ (see Methods for more details). The mean path length is defined as the average shortest path between all possible pairs of nodes on the graph. The clustering coefficient is a measure of the degree of clustering of the nodes on the graph. We calculated these measures for the diverse set of constructs described above and found that the mean path lengths of condensate graphs are only slightly larger than those of Erdős-Rényi graphs^45^ (Fig. 3e), whereas the mean clustering coefficients are three to seven times larger for condensate graphs (Fig. 3f). These features highlight the non-random, inhomogeneous, small-world nature of condensate graphs wherein a few molecules make up hubs in the network, and the rest of the molecules are connected to these hubs via sticker-mediated physical crosslinks.

The observed small-world network structures imply that even within condensates formed by molecules of a single type, the crosslinking density will be inhomogeneous, on average. This can give rise to time-dependent changes of material properties, expected for viscoelastic materials, and physical aging^46^, as has been observed for simple condensates such as those formed by PLCDs^11,47^ and other low complexity domains^48^. Additionally, the type of small-world network that is formed, as defined in terms of the degree, mean path length, and mean clustering coefficient, will be affected by solution conditions (temperature in our case), and the valence and linear patterning of stickers^46^. Our observations that condensate structures fit the description of being graphs that are non-random, inhomogeneous, with a small-world structure on average, might explain why numerous studies based on fluorescence recovery after photobleaching often show the coexistence of slowly recovering or immobile species with rapidly recovering or highly mobile components.

Interestingly, the normalized mean path lengths seem to be independent of temperature- and variant-type. In contrast, the normalized clustering coefficients show some variation, suggesting that the condensate network structure varies with the construct and temperature. As ω approaches 0, the clustering coefficient decreases, in accordance with the increased randomness of the condensate structure as the temperature approaches *T*_c_. We also find that the FUS-LCD clustering coefficient is smaller than that of WT A1-LCD and the -4F-2Y and -10F+7R+12D clustering coefficients are larger than that of WT A1-LCD. Simulations that include the FUS-LCD involve fewer chains than those that include the WT A1-LCD, due to the increased length of the FUS-LCD. These results suggest that decreasing the number of chains (nodes), while maintaining a similar number of sticker-sticker interactions (edges), results in decreased small-world networking, as in the case of FUS, whereas decreasing the number of sticker-sticker interactions, while maintaining the number of total chains, results in increased small world-networking, as in the case of -4F-2Y and -10F+7R+12D. Taken together, we find that the extent of small-world networking is proportional to the ratio of total number of sticker-sticker interactions.

Recently, Shillcock et al.^38^, used a specific implementation of graph-theoretical approaches to analyze their simulations of condensates formed off a lattice for generic sticker-and-spacer models. They concluded that the connectivity of condensate networks is much greater than that of random networks, highlighting the fact that these condensates are more elastic than pure fluids. It is worth noting that the simulations of Shillcock et al.^38^, are of model semi-flexible chains in a good solvent. Under these conditions, segregative transitions such as phase separation cannot be realized^25,49^. Instead, what Shillcock et al., observe and analyze is percolation without phase separation. PSCP generates two coexisting phases, whereas percolation without phase separation is a continuous transition that does not yield two coexisting phases^19^. In this context, it is interesting that Shillcock et al., also find that collecting polymers into a percolated network engenders chain expansion within the clusters when compared to dispersed monomers.

### Molecular features of condensate interfaces

**Fig. 4:**
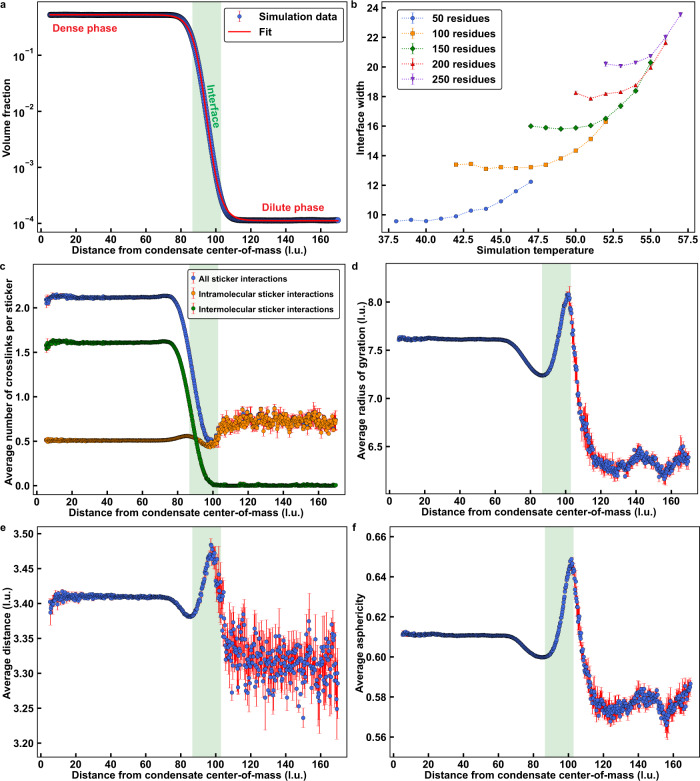
Interfaces of condensates have distinctive conformational characteristics. **a** A representative radial density plot of a simulation of the wild-type A1-LCD at ω = −3.38. The solid red curve corresponds to a logistic fit to the data (see Methods). **b** The width of the condensate interface versus temperature for simulations of homopolymers at different lengths. **c** Average number of sticker-sticker crosslinks per sticker plotted against the distance from the condensate center-of-mass for wild-type A1-LCD at ω = −3.38. Depicted are the total number of crosslinks (blue), the number of intramolecular crosslinks (orange), and the number of intermolecular crosslinks (green). **d** Average *R*_*g*_ of a chain plotted against the distance from the condensate center-of-mass for the wild-type A1-LCD at ω  = −3.38. **e** Average distance between residues on the same chain that are separated by exactly five residues plotted against the distance from the condensate center-of-mass of one of the residues for the wild-type A1-LCD at ω = −3.38. **f** Average asphericity of chains plotted against the distance from the condensate center-of-mass for the wild-type A1-LCD at ω = −3.38. Values of asphericity that are larger than 0.4 point to cigar-shaped conformations, at least on the local level^56^. The distinction of chain dimensions across the dilute, dense, and interfacial regions disappears as the critical temperature is approached. In panels **a**, **c**, **d**, **e**, and **f**, the translucent green rectangles represent the interfacial region as determined by the logistic fit and the error bars signify standard errors about the mean across 3 replicates. In panel **b**, error bars represent standard errors about the mean across 10 replicates. l.u. is lattice units. Source data are provided as a Source Data file.

**Fig. 5:**
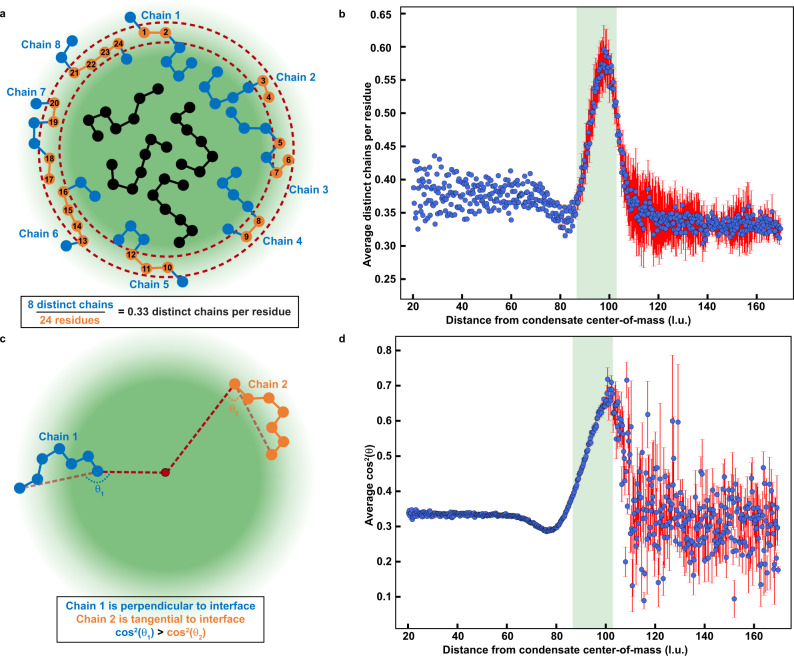
At the interface, molecules have non-random, perpendicular orientations. **a** A diagram depicting how distinct chains per residue is calculated. The region enclosed by the dashed red curves indicates the radial shell of interest. Any chains that contain beads within the radial shell are colored blue. Any beads that are within the radial shell are colored orange. All other chains are colored black. To calculate the distinct chains per residue, the number of blue chains is divided by the number of orange beads, in this case 8 and 24, giving a parameter value of 0.33. This parameter can vary between 0 and 1. Lower values suggest that chains are wrapped around a radial shell, whereas higher values suggests that chains are oriented perpendicular to a radial shell. **b** Average distinct chains per residue plotted against the distance from the condensate center-of-mass for the wild-type A1-LCD at ω = −3.38. **c** A diagram depicting how the parameter cos^2^θ is calculated. Here, θ is defined as the angle swept out by a line segment (translucent dashed red line) between the first and last beads of a chain and a line segment (opaque dashed red line) between one of the beads and the condensate center. Chain 1 (blue polymer) is oriented more perpendicular to the condensate interface. Therefore, θ_1_ is close to 180° and cos^2^θ_1_ ≈ 1. Conversely, chain 2 (orange polymer) is tangential to the interface, such that θ_2_ is close to 90°, and cos^2^θ_2_ ≈ 0. In general, lower values of cos^2^θ suggest that chains are wrapped around a radial shell, whereas higher values suggest that chains are oriented perpendicular to a radial shell. **d** Average cos^2^θ plotted against the distance from the condensate center-of-mass for the wild-type A1-LCD at ω=−3.38. As the critical temperature is approached, the orientational differences across distinct regions vanish. Translucent green rectangles in **b** and **d** represent the interfacial region determined by the logistic fit in Fig. 4a. Error bars signify standard errors about the mean across three replicates and l.u. is lattice units. Source data are provided as a Source Data file.

In the two-phase regime, there exists an interface between coexisting dilute and dense phases. To analyze the condensate interface with statistical robustness, we performed simulations of the WT A1-LCD involving 10^4^ distinct chains (Figs. 4 and 5). This affords us a significantly larger dense phase to analyze. Further, this affords very clear delineations among the dense phase, the interface, and the dilute phase, which we identify by analyzing the radial density profiles (Fig. 4a). Each radial density profile has two shoulders corresponding to coexisting regions of low and high densities. The density in the transition region changes monotonically between the two shoulders. This is the presumed interface between the coexisting dilute and dense phases. The interface will be defined by the wavelength of capillary fluctuations, the sizes of molecules at the interface, the surface density of molecules, and the orientations of molecules with respect to the interface^50,51^. Following precedents for describing liquid / vapor interfaces in van der Waals fluids and associative molecules^52–55^, we use a hyperbolic tangent function^53,55^ to fit the computed radial density profile ϕ(*r*) at a given temperature. The function used is shown in Eq. (3):

3 $${{{{{\rm{log }}}}}}_{10}[\phi (r)]=	\frac{1}{2}\Bigg[{{{{\rm{log }}}}}_{10}(\phi ^{\prime\prime} )+{{{{\rm{log }}}}}_{10}(\phi ^{\prime})\Bigg]-\frac{1}{2}\Bigg[{{{{\rm{log }}}}}_{10}(\phi ^{\prime\prime} ) \\ 	 -{{{{\rm{log }}}}}_{10}(\phi ^{\prime} )\Bigg]\tanh \Bigg[\frac{2(r-{r}_{{{{{{\rm{mid}}}}}}})}{\varDelta }\Bigg];$$

Here, ϕ′ and ϕ″ are the densities in the dilute and dense phases, respectively; *r*_mid_ is the midpoint of the hyperbolic tangent function, and ∆ is the inferred width of the interface. As shown in Fig. 4a, the computed radial density profile can be well described by the hyperbolic tangent function. We used this function to analyze how the width of the interface (∆) scales with chain length (*N*) for homopolymers that were modeled using the parameters obtained to reproduce the measured and computed binodals of the wild-type A1-LCD (Supplementary Fig. 7). The width increases with temperature (Fig. 4b). Further, away from the critical temperature, we observe a plateauing of ∆ to a length-specific value ∆_p_, where ∆_p_ ~ *N*^0.45^. This implies that the width of the interface increases with the increasing molecular weight of the flexible polymer. Above a length-specific temperature, as the temperature approaches *T*_*c*_, the width of the interface (∆), which continues to increase, becomes independent of chain length.

Next, we analyzed the progression of inter-sticker contacts along the radial density profile (Fig. 4c). We observe a monotonic decrease in the average number of intermolecular, inter-sticker interactions along the radial coordinate *r* that progresses from the dense phase into the dilute phase (Fig. 4c). However, the average number of intramolecular, inter-sticker interactions changes non-monotonically. This value, which is low in the dense phase, decreases further through the interface, followed by an increase as *r* extends beyond the interface into the dilute phase (Fig. 4c). The conformational consequences of this non-monotonic change in intramolecular crosslinks per sticker are summarized in Fig. 4d–f. As shown in Fig. 4d, the *R*_*g*_ values of individual molecules are largest within the interface and smallest within the dilute phase. The preference for expanded conformations is also manifest on local length scales as shown in Fig. 4e. Here, we demonstrate that sections of the chain that are up to five bonds long are generally more expanded at the interface when compared to the dense and dilute phases. The global expansion results from more prolate-shaped conformations^56^, as is shown by the evolution of the average asphericity^56^ along the radial coordinate (Fig. 4f). Figure 4d–f also shows a dip in the representative features just to the left of the interfacial peak. This suggests that chains on the condensate side of the interface undergo what we would naively expect: the chain density is lower than in the dense phase, resulting in fewer intermolecular interactions, more intramolecular interactions (Fig. 4c), and slight chain compaction. Alternatively, chains on the solvent side of the interface are more expanded, demonstrating an asymmetry between the inner and outer sides of the interface. Overall, the results in Fig. 4 show that the width of the interface, even away from *T*_*c*_, is approximately three times larger than the average *R*_*g*_ of chains in the dilute phase. This implies that the width of the interface is at least as large as the mean end-to-end distance of a flexible PLCD. This observation is consistent with inferences reported in a recent study by Böddeker et al.^57^, of condensates being defined by thick interfaces.

### Chains are oriented normally at the condensate interface

The increased global and local expansion we observe on average for molecules at the interface raises two possibilities for the orientations of molecules. First, they could be expanded because they adsorb and are oriented parallel to the interface. This arrangement would minimize the number of chains per unit area, ensuring that un-crosslinked stickers at the interface originate from a small number of distinct chains for a given condensate size. Alternatively, the chains could have a locally perpendicular orientation with respect to the interface. This arrangement would maximize the number of distinct chains at the interface while minimizing the number of unsatisfied stickers per chain. We computed the average number of distinct chains per residue (Fig. 5a), resolved along the radial coordinate pointing from the center of the condensate. This value is maximized at the interface (Fig. 5b), implying that molecules do not adsorb, and are not oriented parallel to the interface. Instead, each chain section is oriented perpendicularly to the interface. To further test for this, we computed the projection angles of chain end-to-end vectors with respect to the radius vector with origin at the center of the condensate that is being analyzed (Fig. 5c). Resolved along the radial coordinate, we find that the chains prefer perpendicular orientations at the interface and random orientations within condensates and in the dilute phase (Fig. 5d).

### Unique interfacial features are robust across different PLCDs

We repeated the analyses performed in Figs. 4 and 5 for the diverse set of polymers analyzed in Supplementary Fig. 9b. Temperatures for each construct were chosen *post-facto* to keep the width of the two-phase regime, ω, closest to that of the WT A1-LCD in Figs. 4 and 5 (−3.38), though there are still minor differences in ω values. Our findings are shown in Supplementary Figs. 14–19. In general, we find that our results regarding chain expansion and perpendicular orientations at the interface persist for all constructs, suggesting that these results are robust for polymers with similar architectures such as A1-LCD and the FUS-LCD. While the findings are generally robust, we do observe a few expected differences across constructs. The average number of crosslinks per sticker (Supplementary Fig. 14) is highly dependent on the number of aromatic residues in the sequence. The average number of distinct chains per residue (Supplementary Fig. 18) is lower for longer sequences, such as the FUS-LCD. This is because simulations with FUS have fewer total chains but a similar number of total residues when compared to A1-LCD simulations. As temperature increases, the interface widens, or more precisely, the boundary between the dense and dilute phases becomes less well delineated. Below, we analyze how this loss of delineation comes about.

### The dilute phase crosses over into the semi-dilute regime as *T* approaches *T*_*c*_

We find that on a semi-log scale, the dilute arms of binodals shift rightward with increasing temperature, whereas the dense arms show little change (Supplementary Fig. 2). This implies that the width of the two-phase regime shrinks, and the interface is smeared because of an increase in the saturation concentration with temperature. Note that PLCDs have upper critical solution temperatures^13^. In polymer solutions, there exists a special concentration that equals the concentration of chain units within the pervaded volume of a single chain^58^. This is known as the overlap concentration c* - so named due to the high likelihood that chains will overlap with one another when the solution concentration exceeds c*^59^. In dilute solutions, *c* < c*, whereas in semi-dilute solutions, *c* ≈ c*. We used the mean end-to-end distance values in the single-chain limit^60^ to compute temperature-dependent overlap volume fractions ϕ^*^(*T*) for the wild-type A1-LCD. For temperatures below 20 °C, ϕ_sat_(*T*) < ϕ^*^(*T*) i.e., the left arm of the binodal is located to the left of the overlap line (Supplementary Fig. 11a). Accordingly, for *T* < 20 °C, the dispersed phase that coexists with the dense phase fits the definition of being a dilute solution. However, we observe a crossover above ~20 °C whereby ϕ_sat_(*T*) > ϕ^*^(*T*), which is caused by the increased density within the dilute phase (compare Supplementary Fig. 11b vs. c). Therefore, the dispersed phase that coexists with the condensate is semi-dilute for temperatures above ~20 °C. These distinctions are relevant because the properties of polymer solutions in dilute solutions are governed exclusively by the interplay of intramolecular and chain-solvent interactions. Conversely, the physical properties of semi-dilute solutions are governed by the interplay of density fluctuations and conformational fluctuations, which impacts intramolecular, intermolecular, and chain-solvent interactions^58,59^. The broader implications of this finding become relevant considering recent results highlighting the presence of non-trivial clusters within dilute phases^26^. They are also relevant as a plausible explanation for explaining the observation that motions, as measured using single particle tracking, are not hindered across phase boundaries^61,62^.

## Discussion

We have built upon recent experimental characterizations of phase behaviors of the A1-LCD system and designed variants thereof to develop a lattice-based, single-bead-per-residue model that accurately captures the measured binodals of PLCDs. It is noteworthy that the data of Bremer et al.^13^, and those on unrelated low-complexity domains have also been qualitatively and quantitatively reproduced by other, off-lattice coarse-grained models^63,64^. A specific approach used to compare computations and experiments is through the comparison of computed vs. experimentally derived critical temperatures^63^. However, estimates of *T*_*c*_ are inaccessible from direct measurements. They are instead inferred by fitting binodals extracted from a preferred mean-field theory, cf., Supplementary Fig. 11a. Then, a specific functional form for the width of the two-phase regime^35,63,65^ is fit to the entire binodal. However, based on the Ginzburg criterion, the functional form that is routinely used^35,63,65^ is only valid in the vicinity of the critical temperature^66^. As discussed alongside Fig. 2c, it is unclear how close one needs to be to the critical temperature for this functional form to be valid. Here, we pursue a different approach to compare computed and experimentally derived phase diagrams. Specifically, we quantify the ERMSL between computed and measured low-concentration arms of binodals. We focused on the low-concentration arms because they change the most with temperature and have smaller error bars in experimental measurements when compared to concentrations corresponding to the high-concentration arms of binodals. Overall, the ERMSL values indicate that maximal deviations are a factor of 2-2.5 across concentrations that vary by at least three orders of magnitude. Encouraged by the accuracy of our simulations across 31 different variants of the A1-LCD system plus the FUS-LCD, we used the computed ensembles within, outside, and at the interface of condensates to investigate molecular and mesoscale structures.

**Fig. 6:**
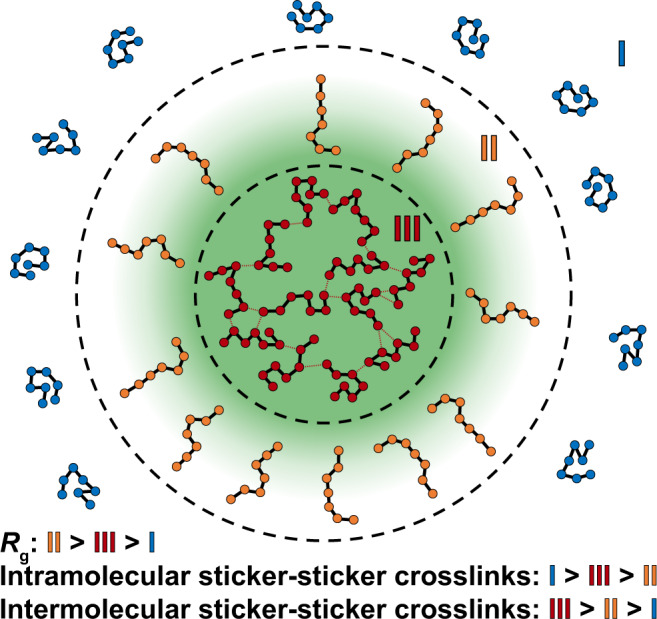
Molecular properties of interfaces are distinct from dilute and dense phases. Diagram summarizing our findings concerning condensate organization. Region I is the dilute phase, Region II is the condensate interface, and Region III is the interior of a condensate. Region I is characterized by relatively compact chains that form few intermolecular contacts. Region II is characterized by relatively expanded chains that are oriented perpendicular to the interface and form the fewest number of total sticker crosslinks. Region III is characterized by chains that are less compact than those in Region I and less expanded than those in Region II. These chains form numerous intermolecular sticker crosslinks, giving rise to a small-world percolated network.

Our findings regarding the degree of crosslinking and extent of chain expansion in the three regions, viz., dilute phase (I), condensate interface (II), and condensate interior (III), are summarized in Fig. 6. Our results suggest that interfaces between condensates and the coexisting dilute phases should be thought of as being thick^57^ rather than thin. This feature of the interface is realized by the ability of disordered proteins to be relatively more expanded, both locally and globally, when compared to the dilute and dense phases. It is known that the interfacial tension decreases as the inverse square of the size of the molecule^50^. Accordingly, the low interfacial tensions that have been measured to date^47,67–69^ appear to originate from chains being most expanded as they traverse the interface. Importantly, the interface features a high number of unsatisfied stickers, achieved due to the high number of chains that project perpendicularly to the interface.

Our observations regarding interfaces have two implications. First, although the number of unsatisfied stickers per chain is minimized, the presence of a high absolute number of unsatisfied stickers, typically defined by the presence of functional groups, suggests that interfaces might be prime locations for enhancing the efficiencies of biochemical reactions that are influenced by condensate formation. This speculation, based on the features we have documented for interfaces, is consistent with numerous observations from the microdroplet literature^70^. Second, it is conceivable that interfaces can catalyze amyloid fibril formation through secondary nucleation^71,72^. This proposal is based on the preference for the high likelihood of accessing locally extended, β-strand-like conformations for molecules such as A1-LCD or mutants of such systems^11^. Our proposal appears to be supported by the recent results of Linsenmeier et al.^73^, who report that amyloid formation is nucleated at condensate interfaces.

Overall, our findings suggest that even the simplest condensates, formed via effective homotypic interactions among PLCDs with sticker-and-spacer architectures, have complex internal structures and interfacial characteristics. The features we have identified are likely to be germane to recent discoveries that condensates are in fact, viscoelastic network fluids^74,75^. We find that condensates formed by PLCDs have small-world network structures. This implies that the molecules are organized into inhomogeneous networks within condensates defined by regions of high vs. low crosslinking densities. The clustering of stickers within condensates, achieved via strong and specific inter-sticker interactions, can be separated from the contributions of spacers that directly impact the solvation preferences, thereby modulating the locations of the dilute arms of binodals. The extension of our findings to multicomponent, multiphasic systems^76^ will be of considerable ongoing and future interest.

## Methods

### Monte carlo simulations using LaSSI

Details of LaSSI simulations and the development of the sticker-and-spacer model can be found in the Supplementary Methods. All sequences used in this study can also be found in Supplementary Table 2.

### Measuring the phase behavior of the FUS-LCD

The two-phase coexistence curve (binodal) of the FUS-LCD was measured as described in Bremer et al.^13^

### Analysis of conformational properties in dense and dilute phases

In Figs. 2 and 3, Supplementary Figs. 5 and 6, we report conformational characteristics of PLCD molecules in dense and dilute phases. To perform these analyses, we first ensure that our simulation shows stable phase separation into a single, distinct dense phase. We then determine whether a chain belongs to the dense or dilute phase based on whether it is within the interacting range of the largest cluster of chains. If so, we group this chain into the dense phase. Otherwise, we group this chain into the dilute phase.

*Swelling ratio*: In Fig. 2 and Supplementary Fig. 5, we introduced the swelling ratio α, which for a given temperature or ω-value, we define as:

4 $$\alpha=\frac{\sqrt{\langle {R}_{g,{{{{{\rm{dense}}}}}}}^{2}\rangle }}{\sqrt{\langle {R}_{g,{{{{{\rm{dilute}}}}}}}^{2}\rangle }}$$

As noted in the main text, we defined the width of the two-phase regime in terms of the parameter ω. Rather than directly calculating the difference between the dense and dilute phase concentrations, which is heavily biased by the dense phase concentration, we calculate the difference between the concentrations on a log scale. This accounts for the fact that the dense and dilute phase concentrations differ by orders of magnitude. The swelling ratio quantifies the degree of expansion of chains in the dense phase relative to the dilute phase. In Fig. 2c, we fit the following exponential decay model to data for the swelling ratio:

5 $$\alpha=1+{{{{{\rm{exp}}}}}} [-a(\omega -b)]$$

Here, *a* is the fitted parameter that controls the rate of decay, and *b* shifts the curve to the left or the right. The parameters for the master curve shown in Fig. 2c are *a* = 0.33, and *b* = −9.5, respectively.

### Ternary plots to analyze the interplay of intra-chain, inter-chain, and chain-solvent interactions in the dense phase

Supplementary Fig. 6 shows a ternary plot constructed in the following way: (1) For every chain in the dense phase, we calculate the *R*_*g*_ values of individual molecules. (2) For each bead in that chain, we count the number of neighbors within $\sqrt{3}$ lattice units that are empty (i.e., solvated), contain beads that belong to the same chain, or contain beads that belong to other chains. (3) Sum each count for every bead in the chain and divide by the sum of the three counts. This yields the three fractions, *f*_sol_, *f*_intra_, and *f*_inter,_ respectively. (4) For each chain, we then determine which bin it belongs to on the ternary plot (based on the fractions) and average *R*_*g*_ calculated for all chains in that bin to determine the color of the bin of interest.

### Overlap concentration calculation

In Supplementary Fig. 11a, we calculate the overlap concentration of simulated constructs using the method of Wei et al.^60^. Specifically, we use the following equation:

6 $$\phi \ast=\frac{N{r}^{3}}{{\left(\sqrt{\langle {R}_{e}^{2}\rangle }\right)}^{3}}.$$

Here, ϕ* is the overlap concentration, *N* is the number of monomers in a chain, *r* is the radius of individual residues and $\sqrt{\langle {R}_{e}^{2}\rangle }$ is the root mean square end-to-end distance of a chain. In our case, *N* is 137 and *r* is set to 0.5 lattice units. We apply this equation to chains in the dilute phase to minimize the effects of intermolecular interactions on the calculation of the overlap concentration dictated purely by conformational fluctuations.

### Parameterization of a model for an equivalent homopolymer

In Supplementary Fig. 7, we introduce a homopolymer equivalent for the wild-type A1-LCD. The contact energies for this model were parameterized by choosing a single pairwise interaction energy such that the phase diagrams of the homopolymer and wild-type A1-LCD overlay on one another. A pairwise interaction energy of −3.3 accomplishes this task.

### Graph theoretical analyses

In Fig. 3 and Supplementary Figs. 9, 10, 13, we report on the small-world network structure formed by condensates. For this, we analyze the undirected, unweighted graphs formed by each condensate over the equilibrated portion of the trajectory^44^. For each snapshot, we associate the condensate with the largest connected number of chains. Two chains are considered connected if at least one pair of stickers between them are adjacent, defined by being within $\surd 3$ lattice units on the cubic lattice. We also calculate the betweenness centrality of each chain as defined in Eq. (2). For each condensate, we calculate the average path length and the average clustering coefficient to verify the small-world characteristics of the graph. The empirical average path length, representing the average number of steps along the shortest paths for all possible pairs of nodes (*v*_*i*_, *v*_*j*_), is calculated according to:

7 $$L=\frac{2}{n(n-1)}\mathop{\sum}\limits_{\begin{array}{c}i,j=1,n\\ i\ne j\end{array}}d({v}_{i},\,{v}_{j})$$

Here, *n* is the number of nodes. We compare this value to the average path length assuming Erdős-Rényi statistics^77^, which we calculate as $\frac{{{{{\rm{log}}}}}\,{{{{\rm{n}}}}}}{{{{{{\rm{log}}}}}}{{\langle {k}_{v}\rangle }}}$ for each condensate, where $\langle {k}_{v}\rangle$ is the average degree. Finite-size effects were not accounted for because $\langle {k}_{v}\rangle < < n$. We calculate the global clustering coefficient following the work of Watts and Strogatz^78^ by averaging over the local clustering coefficients for all nodes. For an undirected graph, the local clustering coefficient is given by:

8 $${C}_{i}=\frac{2|\{{e}_{jk}:{v}_{j},\,{v}_{k}\in {n}_{i},\,{e}_{jk}\in E\}|}{{k}_{v,i}({k}_{v,i}-1)}$$

This calculation applies to nodes *v*_*j*_ and *v*_*k*_ that are in the neighborhood of node *n*_*i*_, with *e*_*jk*_ edges, in the set *E* of edges. The Erdős-Rényi value is calculated for each snapshot as $\frac{\langle {k}_{v}\rangle }{n}$.

### Analysis of internal organization of condensates

In Supplementary Fig. 8a–c, we report the likelihood that a sticker within a condensate is a neighbor of another sticker vs. a spacer, normalized by the same likelihood for the homopolymer. We calculate this parameter in the following way: (1) For a given condensate, we go through all the beads in each of the chains. (2) If a bead is a sticker (Tyr or Phe), we tally the number of its neighbors that are within $\sqrt{3}$ lattice units that happen to be stickers (*n*_st_) and the number of neighbors that are spacers (*n*_sp_). (3) We sum over all values to calculate *p*_a,seq_ (see main text). (4) We repeat steps 1-3 for the homopolymer condensate to calculate *p*_a,ref_. For this calculation, we assume that the homopolymer has the same sticker-spacer architecture as the wild-type A1-LCD. (5) The ratio of association *g*_a_ is then computed as shown in the main text. We note that Panel A and Panel C use wild-type A1-LCD (WT) as the background for the homopolymer, whereas Panel B uses WT + NLS, which includes one extra Tyr residue compared to WT.

### Fitting Weibull distributions to sticker cluster probability distributions

In Supplementary Fig. 8d–f, we analyze the probability distributions for realizing clusters with stickers that form via inter-sticker crosslinks. For each equilibrated snapshot, we calculate the relative frequency that stickers form a cluster of a particular size, where the size is determined by the total number of stickers in the cluster. We then multiply each frequency by the cluster size to obtain the probability for a sticker to be in each cluster. A least-squares analysis, weighted by the inverse of the variance of repeated measurements, was performed on the linearized form of Eq. (1). The analysis was restricted to the linear region of the plot. Outliers, where the distribution was exponentially bounded or where there were limited statistics at large *s* were treated as being a point that is greater than 3 scaled median absolute deviations from the median and hence removed from the analysis. The data treatment was insensitive to different outlier criteria. The values for the Weibull parameters were extracted directly from the fits to the linearized form of the cumulative distribution function given by Eq. (1).

### Analysis of radial features to determine radial bins

Figures 4, 5, and Supplementary Figs. 11, 14–19 contain analyses of radial features of simulations. For each analysis, we use radial shells with thickness 1/4 of a lattice unit for the purpose of binning values together. In cases where we need a prior radial distribution, namely for determining the volume fraction in Fig. 4a and Supplementary Fig. 11b, c, we use the exact prior for a cubic lattice with side-length 120. When calculating the radial bins for the chains in Fig. 4d, f, and Supplementary Figs. 15, 17, rather than using the center-of-mass of a chain and counting each chain one time, we independently count each bead in the chain using the radial bin of the bead and the radius of gyration (Fig. 4d and Supplementary Fig. 15) or asphericity (Fig. 4f and Supplementary Fig. 17). This accounts for the fact that a single chain can span multiple bins by weighting each bin based on how many of a chain’s beads belong to it. Alternatively, in Fig. 4e and Supplementary Fig. 16, we only use the first bead in determining the radial bin.

### Average number of crosslinks per sticker

In Fig. 4c and Supplementary Fig. 14, we calculate the average number of crosslinks per sticker. To do so, we go through every sticker, defined as a Tyr or Phe residue, in the system and count how many of its neighbors within $\sqrt{3}$ lattice units are also stickers. Each neighboring sticker represents one inter-sticker crosslink. We also determine whether each of these crosslinks is an intra- or intermolecular crosslink.

### Average distinct chains per residue

In Fig. 5a, b and Supplementary Fig. 18, we describe and report a parameter which we term “average distinct chains per residue.” To calculate this parameter, we do the following: (1) for each radial shell, we count the number of distinct chains with beads that are contained in this shell. (2) Designate the number of distinct chains *n*_*c*_ and the total number of beads in the shell *n*_*b*_. (3) Calculate the final parameter as *n*_*c*_ / *n*_*b*_. This parameter is necessarily between 0 and 1. Lower values suggest that the beads in the given radial shell belong to a few distinct chains, whereas higher values suggest that the beads belong to many distinct chains.

### Orientational analysis

In Fig. 5c, d and Supplementary Fig. 19, we describe and report a parameter that describes chain orientation relative to the condensate center-of-mass. To calculate this, we do the following: (1) For each chain in the system, we consider a line segment drawn from one end of the chain (bead *a*) to the other (bead *b*). (2) Next, we consider the line segment drawn from bead *a* to the condensate center-of-mass. (3) We then label the angle swept out by the line segments in steps 1 and 2 as *θ*. (4) Calculate cos^2^ *θ* and the radial bin to which *a* belongs. (5) We repeat steps 2-4 using bead *b* instead of bead *a*. (6) For each radial bin, average the associated cos^2^ *θ* values. This parameter is necessarily between 0 and 1.

### Reporting summary

Further information on research design is available in the Nature Portfolio Reporting Summary linked to this article.

## Supplementary information

Supplementary Information

Reporting Summary

## Source data

Source Data

## Data Availability

All simulation results and the details for reproducing the analyses are available via the GitHub repository of the Pappu lab: https://github.com/Pappulab/Interfacial_Analyses. Raw simulation data are available upon request.bSource data used to generate all figures are provided with this paper. Source data are provided with this paper.

## Code availability

All code used in this work is available via the GitHub repository of the Pappu lab: https://github.com/Pappulab/Interfacial_Analyses. Farag, M., Cohen, S., Borcherds, W., Bremer, A., Mittag, T., & Pappu, R. (2022). Condensates formed by prion-like low-complexity domains have small-world network structures and interfaces defined by expanded conformations. Code for interfacial_analyses available at: 10.5281/zenodo.1234.
