# Supplementary material for: Condensates formed by prion-like low-complexity domains have small-world network structures and interfaces defined by expanded conformations
Source: Nat Commun. 2022 Dec 13;13:7722. doi: 10.1038/s41467-022-35370-7 (PMC9748015; doi:10.1038/s41467-022-35370-7)
Supplement: Supplementary file 1 — Supplementary Information [file 41467_2022_35370_MOESM1_ESM.pdf]

Supplemental Material for:

Condensates formed by prion-like low complexity domains have small-world network structures and interfaces defined by expanded conformations

Mina Farag[1], Samuel R. Cohen[1], Wade M. Borcherds[2], Anne Bremer[2], Tanja Mittag[2], Rohit V. Pappu[1*]

[1]Department of Biomedical Engineering and Center for Biomolecular Condensates (CBC), Washington University in St. Louis, St. Louis, MO, USA

[2]Department of Structural Biology, St. Jude Children's Research Hospital, Memphis, TN, USA

*e-mail: pappu@wustl.edu

Supplementary Methods

Details of LaSSI simulations

Monte Carlo simulations using LaSSI: Coarse-grained simulations were performed using LaSSI[1], a lattice-based Monte Carlo simulation engine that is based on the generalized bond fluctuation model. The coarse-grained model uses one lattice bead per amino acid residue and involves a pseudo-implicit solvent model in that a vacant lattice site is solvent. In this way, solvent is afforded space in the system, but we do not parameterize any explicit interactions between solvent occupied sites and any other sites. Accordingly, the solvent entropy is explicitly accounted for, albeit in a solvent non-specific manner, but the energetics are implicit. This enables fast simulations with $O(10^4)$ interacting beads and $O(10^2)$ molecules, allowing us to access the mesoscale features of biomolecular condensates.

Monte Carlo moves are accepted or rejected based on the Metropolis-Hastings criterion such that the probability of accepting a move is the $\min[1,\exp(-\Delta E/k_BT)]$ where ΔE is the change in total system energy of the attempted move and k_BT is the thermal energy. Total system energies were calculated using a nearest neighbor model whereby any two beads that are within one lattice unit of each other along all three coordinate axes contribute to the total energy of the system. In our case, we define all pairwise interaction energies as absolute energies (Fig. 1a) that are scaled by the simulation temperature during the Metropolis-Hastings step. The full set of Monte Carlo moves used in each type of simulation performed in this work are shown in Supplementary Table 1. Most moves are fully described in Choi et al., [1]. Here, we describe the moves used in this work that were developed after the initial release of LaSSI and are therefore not previously described. The "multi-local" move attempts to move a single bead and its covalently bonded partners by -1, 0, or 1 lattice unit along each coordinate axis. The "pivot" move chooses a random chain, then chooses a random bead, *x*, along this chain. The move attempts to pivot every bead in the chain beyond *x* in the same direction by 90°.

Calculation of coexistence curves and interfacial features: Multi-chain LaSSI simulations at various temperatures were performed to calculate coexistence curves. To speed up condensate formation, we initialized the system in a relatively concentrated state. Specifically, all simulations discussed in this work used a 120×120×120 cubic lattice with periodic boundary conditions. However, the simulations were initialized in a smaller 35×35×35 cubic lattice. This method allows for significantly faster equilibration processes, but precludes the analysis of pre-equilibrium processes, such as condensate coalescence. For each variant, 200 chains, each with 137 beads, were placed in the cubic lattice. Thus, the total volume fraction of beads was approximately 0.016.

Simulations involving the FUS-LCD, which is 216 residues long in our case, included 128 chains. This matches the bulk volume fraction achieved using the A1-LCD system. Simulations involving homopolymers with length 50, 100, 150, 200, and 250 included 600, 300, 200, 150, and 120 chains, respectively.

Multi-chain simulations were performed with 1.5×10^{10} steps. Simulations typically equilibrated after about 2×10^{9} steps, as determined by a plateauing of the total system energy. To be conservative, all simulation results were analyzed after the halfway point of 7.5×10^{9} steps (Supplementary Figure 20). For systems comprising associative polymers, there are two distinct types of phase transitions that are realizable. These are the segregative transition *viz*., phase separation, and the associative networking transition or percolation. Percolation, computed simply as the size or number of molecules in the single largest cluster, is often conflated with the order parameter for phase separation, which requires the detection of a density transition. To avoid this conflation, we compute two distinct order parameters in the LaSSI paradigm. Our analysis of percolation matches that used in the literature and introduced in the context of condensate simulations by Harmon et al., [2]. For identifying the presence of a phase boundary, we computed radial distribution of beads in the system as described in the original work of Choi et al., [1]. Eq. (2) of the main text was fit to the radial distribution, resulting in estimates for dilute phase concentrations, dense phase concentrations, interface midpoints, and interface widths. Multi-chain simulations of the WT A1-LCD and homopolymers were performed with 10 replicates, with each simulation initiated by a distinct random seed.

Multi-chain simulations of the A1-LCD variants and the FUS-LCD were performed in triplicate, each simulation being initiated by a distinct random seed. All sequences used in this work are shown in Supplementary Table 2. In the case of A1-LCD variants, these were taken from the work of Bremer et al., [3]. In all cases, the calculated volume fractions were near identical across replicates. At low temperatures, the calculated dilute phase volume fractions tend to have more variability due to the decreased likelihood that chains are in the dilute phase. To convert from simulation temperature and volume fraction to experimental temperature (in Kelvin, then shown as degree-Celsius in the results) and volume fraction, fixed scaling factors of 5.6 and 0.6 were used, respectively. These scaling factors, which are based on those used by Martin et al.,[4] were used for all variants (Supplementary Figure 2). In general, scaling factors are required to convert computed binodals to the currency of experimental measurements. To convert from volume fraction to mass concentration, we assumed that a volume fraction of 1.0 corresponds to a mass concentration of 1310 mg/ml as prescribed by Wei et al., [5].

In Figs. 4-5, we report the interfacial features of condensates. To increase the size of the dense phase, and to delineate the dense phase, the interface, and the dilute phase, we performed simulations involving 10^{4} chains. These simulations were significantly more computationally expensive than the prior 200 chain simulations, and thus were not performed for any chains other than the WT A1-LCD. These simulations were performed in triplicate, each with 5×10^{11} steps, and were analyzed after 3.5×10^{11} steps.

Assessments of chain dynamics: In Supplementary Figure 13, we present an analysis of the dynamics of chains within condensates. For these analyses, we performed simulations using only local moves (see Supplementary Table 1). The results are presented in terms of the number of Monte Carlo steps, which we use as a proxy for time. This approach is reasonable and only applicable when moves of a certain flavor are deployed to the exclusion of all other moves.

Because these simulations include only local moves, we perform simulations for a total of 10^{12} steps. The simulations were analyzed once every 2.5×10^8 or 2.5×10^9 moves, with only the second half of the simulation being used in the analysis. To quantify errors, we performed five independent simulations for each simulation temperature. These independent simulations were initiated using distinct random seeds, and small error bars, computed across the independent replicates, affirm the robustness and reproducibility of our results. Other simulation conditions were the same as for other multi-chain simulations described previously. Simulations designed to assess chain dynamics were performed only for the WT A1-LCD. To interpret the number of Monte Carlo moves on a relevant timescale, we determined the average lifetime of sticker-sticker interactions within the simulations. To do so, we created a list of sticker-sticker interactions at a given timepoint. We compared this list to the same type of list at a later timepoint and counted how many of the same sticker-sticker interactions persisted. Our results, shown in Supplementary Figure 12, demonstrate that sticker-sticker interactions rarely persist for longer than our shortest time-step, 2.5×10^8 Monte Carlo moves, even at the lowest temperature studied, suggesting that our results shown in Supplementary Figure 13 are using a timescales much longer than the average lifetime of a sticker-sticker interaction.

Sticker-and-spacer model development and validation

Our model incorporates distinct interaction energies and a mean-field electrostatic term that explicitly accounts for various amino acids and combinations thereof. We developed our model with the intention of incorporating the most important independent interactions that govern the phase behavior of A1-LCD, while minimizing the risk of over-parameterization. Below, we describe in detail how we developed and validated our sticker-and-spacer model.

Gaussian process Bayesian optimization (*GPBO*) *for parameterization and verification of sticker-and-spacer model*: Single-chain LaSSI simulations at a single temperature ($k_BT = 50$, $k_B = 1$) were performed to parameterize the initial sticker-and-spacer model. **Values of the apparent scaling exponent (ν_{app}) derived from experimental SEC-SAXS data of the variants in Supplementary Table 2 with a single asterisk were used as the target values.** These data come directly from Bremer et al[2]. Values of ν_{app} were calculated as described by Meng et al., [6] using five independent simulations per construct, each entailing 6×10^9 Monte Carlo steps. A Gaussian process Bayesian optimization [7] was implemented as described in Supplementary Figure 1. This process was iterated over pairwise interaction energies to minimize the sum of the square residuals of computed and experimentally derived values for ν_{app} (Fig. 1b). If a given parameter remained close to the upper or lower bound through each iteration, then the parameter bounds used in the optimization were manually changed over the course of the optimization. Over 500 iterations were performed. The final parameters as well as the upper and lower bounds and values are shown in Supplementary Table 3. Here, "Aro" represents either a Tyr or Phe residue and "X" represents any amino acid residue that is not explicitly accounted for. These parameters form the foundation of our coarse-grained model and were chosen based on prior work in Martin et al.,[4] and Bremer et al.,[3] where it was shown that in the context of A1-LCD: (1) aromatic-aromatic interactions were the major determinant of phase behavior; (2) the identity of aromatic residues had a strong effect on aromatic-aromatic interactions and a weak to negligible effect on aromatic-Arg interactions; (3) increasing the number of Lys residues strongly increased the saturation concentration. We note that these findings are almost certainly context dependent. For example, sequences with a larger fraction of charged residues may show a much stronger correlation between the identity of aromatic residues and the strength of aromatic-Arg interactions. We also note that for every

parameter, the difference between the final value and either of the bounds is at least 20% of the absolute final value, suggesting the bounds are adequately distanced.

A mean-field model to account for protein charge: We have previously shown that small changes to the net charge per residue (NCPR) have little effect on single chain dimensions but can alter dilute phase concentrations by orders of magnitude in a predictable manner [3]. Specifically, we developed a mean-field analytical model that accurately captured this difference. Here, we extend our analytical model to account for this electrostatic effect in our computational model by incorporating a mean-field NCPR-based term into our multi-chain simulations. This term strengthens or weakens all interactions of a system depending on the NCPR of the variant. In this way, we implicitly account for ionizable residues without explicitly modeling them as unique, highly solvated spacers. The mean-field term is incorporated as follows:

$$E^* = \frac{E}{1 + a\left|\mathrm{NCPR} - \mathrm{NCPR}_{\mathrm{mid}}\right| + b}; \tag{1}$$

Here, E^* is the new effective pairwise energy, E is the original pairwise energy, NCPR is the net charge per residue of the variant of interest, and $\mathrm{NCPR}_{\mathrm{mid}}$, as well as a and b are constants. The mean-field term can be visualized as an absolute-value function whose minimum vertex is at ($\mathrm{NCPR}_{\mathrm{mid}}$, b) and whose slope is $-a$ to the left of the minimum and $+a$ to the right of the minimum (Supplementary Figure 21). Based on prior work, $\mathrm{NCPR}_{\mathrm{mid}} = 0.0287$, $a = 1.54$, and $b = -0.045$. This ensures that $E^* = E$ for the wild-type A1-LCD. In this way, we have used efficient single-chain simulations coupled to a GPBO method to parameterize interaction strengths, as well as a mean-field electrostatic term to modulate these interaction strengths for multi-chain simulations where ionizable residues play a larger role in driving condensate formation. The major advantages of this decision include a reduced risk of model over-parameterization, zero risk of misrepresenting explicit electrostatic interactions among ionizable residues, water, and solvent ions, more efficient computations, and a simpler overall model. The main limitation of this decision is that we are unable to provide microscopic insights regarding the effects of electrostatic interactions. However, the IDRs studied in this work, namely PLCDs, are relatively charge-depleted, suggesting that these microscopic interactions would play a minor role in the overall condensate organization. We believe that an accurate model of proteins with a high fraction of charged residues would likely require a more explicit account of ionizable residues and of solution ions. We discuss the accuracy of our sticker-and-spacer model with and without the mean-field electrostatic term below.

Parameterization of spacer interactions: Although we did not have SAXS data for the variants designated as "spacer variants" by Bremer et al.,[3] (see variants with a double asterisk in Supplementary Table 2), we were able to parameterize interactions between spacers and all other beads by titrating five new interaction parameters to match the computed binodals to the binodals derived from experiments as shown in Supplementary Figure 2f-i. Parameters for the final model are shown in Fig. 1a and Supplementary Table 4. Ser-X, for example, indicates the pairwise interaction energy between serine and any other residue. In cases where a pairwise interaction is ambiguous (for example, the interaction between Thr and Ser), the weaker interaction is used (in this case, -2.35). Our final sticker-and-spacer model explicitly or implicitly accounts for every amino acid found in the WT A1-LCD except for methionine, alanine, and proline, whose interactions are the only ones governed by the "X-X" parameter. However, within WT A1-LCD, there is one methionine residue, three alanine residues, and three proline residues. Given the relative paucity of these residues and the fact that they are not expected to contribute strongly to

the phase behavior of A1-LCD, we did not deem it necessary to account for these three residues explicitly.

Quantifying the deviations between computed and measured phase boundaries: We evaluate the deviations between LaSSI-derived and experimentally derived phase boundaries using a multi-step process. For each variant, we first perform a linear regression of temperature vs. log(c_{sat}) for the LaSSI-derived dilute arm. Second, for each experimentally-derived data point along the dilute arm, we calculate $x = (c_{\text{sat,sim}} / c_{\text{sat,exp}})$ where $c_{\text{sat,exp}}$ is the experimentally-measured saturation concentration at a specific temperature and $c_{\text{sat,sim}}$ is the estimated c_{sat} from the regression in the first step at the same temperature. Third, we calculate the exponential root mean square log (ERMSL) of x as $\exp\left(\sqrt{\left\langle \left[\ln x\right]^2 \right\rangle}\right)$. The ERMSL is a positive value greater than or equal to 1 and can be interpreted as a measure of the error between the computed and experimentally derived phase boundaries, specifically the dilute arms. For example, an ERMSL value of 10 indicates that, on average, the two c_{sat} values differ by about a factor of 10, or one order of magnitude. Alternatively, an ERMSL value of 1 indicates that there is perfect agreement between the computed and measured dilute arms. The ERMSL is akin to the root mean square log error (RMSLE) often used in machine learning [8], except for two important distinctions: (1) When calculating the RMSLE, 1 is added to both the numerator and the denominator of the argument. In our case, we do not need to include this bias since c_{sat} values are always greater than zero. (2) Unlike with the RMSLE, we take the exponential of our final value to bring our error back to an interpretable scale. This exponential operator is a reciprocal function of the inner logarithmic operator, in the same way that the outer square root operator is a reciprocal function of the inner square operator. By using a linear regression of the simulation-derived dilute arm in the first step, we can account for experimental data at any temperature, regardless of whether we have explicitly performed a simulation at that temperature. We report the ERMSL of every variant in Fig. 1d. This includes the variants in Supplementary Figure 2j, which were not used in the parameterization of the sticker-and-spacer model. We find that most of the variants studied have an ERMSL < 2, and that the maximum ERMSL is ~3.5, suggesting strong quantitative agreement between simulations and experiments.

We tested the effects of our mean-field NCPR-based term by comparing ERMSL values from simulations with and without the term. We note that any variant with an NCPR close to that of the WT A1-LCD will be unaffected by this term. Therefore, we focused on the 12 variants in this study with a different number of charged residues than WT A1-LCD for this analysis. Our results are summarized in Supplementary Figure 22. We find that without the mean-field term, the average ERMSL is about 3.4, whereas inclusion of the term decreases the ERMSL by 42% to about 2.0, suggesting a sizeable increase in the accuracy of our model with the mean-field NCPR-based term. Overall, our final parameterization contains the fewest number of distinct, major interactions, while also affording good agreement with experimental measurements. In addition, the major interactions we have chosen to be parameterized appear to be most important in terms of governing the phase behavior of the set of PLCDs studied here.

Supplementary Discussion

Swelling ratio

We define the swelling ratio as the ratio of dimensions in the dense vs. the dilute phase. Analysis of the simulation results suggests that there is a single master curve to describe the

evolution of the swelling ratio as a function of the width of the two-phase regime, which is our proxy for temperature or solvent quality. Essentially, the swelling ratio is a measure of the difference in the solvent quality across the phase boundary as the temperature approaches T_c.

We obtain the following relationships for the swelling ratio:

$$\alpha_T = \left[\frac{\sqrt{\langle R^2_{g,\text{dense}} \rangle}}{\sqrt{\langle R^2_{g,\text{dilute}} \rangle}} \right]_T ; \tag{2}$$

We define the width of the two-phase regime as:

$$\omega_T = \log_{10}\left[\frac{c_{\text{dilute}}(T)}{c_{\text{dense}}(T)} \right] = \frac{1}{2.303} \ln\left[\frac{c_{\text{dilute}}(T)}{c_{\text{dense}}(T)} \right]; \tag{3}$$

The master curve we obtain is of the form:

$$\alpha_T = 1 + \exp\left[-a(\omega_T - b) \right]; \tag{4}$$

Here, a = 0.33 and b = –9.5. Rearrangement of (3) using the relationship in (2), yields the following:

$$\alpha_T - 1 = \exp(-a\omega_T)\exp(ab) = A_0 \exp(-a\omega_T);$$

$$\frac{(\alpha_T - 1)}{A_0} = \exp(-a\omega_T) = \exp\left[-\frac{a}{2.303} \ln\left(\frac{c_{\text{dilute}}(T)}{c_{\text{dense}}(T)} \right) \right];$$

$$\frac{(\alpha_T - 1)}{A_0} = \exp\left[\ln\left(\frac{c_{\text{dilute}}(T)}{c_{\text{dense}}(T)} \right)^{-\frac{a}{2.303}} \right]; \tag{5}$$

$$\Rightarrow \frac{(\alpha_T - 1)}{A_0} = \left(\frac{c_{\text{dilute}}(T)}{c_{\text{dense}}(T)} \right)^{-\frac{a}{2.303}} = \left(\frac{c_{\text{dense}}(T)}{c_{\text{dilute}}(T)} \right)^{\frac{a}{2.303}} = \left(\frac{c_{\text{dense}}(T)}{c_{\text{dilute}}(T)} \right)^{0.14};$$

Final rearrangement yields,

$$\alpha_T = 1 + A_0 \left(\frac{c_{\text{dense}}(T)}{c_{\text{dilute}}(T)} \right)^{0.14} = 1 + 0.04 \left(\frac{c_{\text{dense}}(T)}{c_{\text{dilute}}(T)} \right)^{0.14}; \tag{6}$$

Here, 0.14 ≈ (0.33/2.303), and $A_0 \approx 0.04$. The measurements and computed binodals show that as T approaches T_c, c_{dilute} approaches c_{dense}. We also note that c_{dense} changes minimally with T and that c_{dilute} changes appreciably as T approaches T_c. The swelling ratio approaches unity as T approaches T_c, and this is evident in the relationship that emerges from the master curve. We can use the measured and computed information regarding the T-dependence of c_{dilute} vs. c_{dense} and simplify the relationship in (6) as:

$$\alpha_T \approx 1 + \frac{0.04\left(c_{\text{dense}}\right)^{0.14}}{\left[c_{\text{dilute}}\left(T\right)\right]^{0.14}}; \tag{7}$$

The implication is that as the overall solvent quality improves, characterized by increasing T in a system showing UCST phase behavior, the improvement in the overall quality translates primarily to an improvement of solvent quality in the dilute phase. Given the behavior we observe, this can be interpreted as the solvent quality in the dilute phase improving to become akin to that of the solvent quality in the dense phase. Importantly, below T_c, the implication is that the apparent solvent quality in the dense phase is *always* better than that of the dilute phase, a manifestation of chains solvating one another in the dense phase, and that the dilute phase catches up with the dense phase monotonically, not in stages as one would expect of crossover behavior. This master curve also provides insights on how the critical temperature should be estimated for finite-sized heteropolymeric systems, whereby T_c should be the temperature at which the swelling ratio is unity.

Supplementary Table 1: Monte Carlo move sets for different types of simulations.

Move	Multi-chain simulation probability	Single-chain simulation probability	Dynamics simulation probability
**Local**	0.51	0.67	1
**Multi-local**	0.17	0.22	0
**Snake**	0.03	0	0
**Translation**	0.03	0	0
**Pivot**	0.09	0.11	0
**Double pivot**	0.17	0	0

Supplementary Table 2: Protein constructs and their associated sequences. Constructs marked with a single asterisk were used in the Gaussian process Bayesian optimization. Constructs marked with a double asterisk were used to manually parameterize spacer residue energies. Unmarked constructs were not used to parameterize the model.

Construct	Amino acid sequence
WT A1-LCD*	GSMASASSSQRGRSGSGNFGGGRGGGFGGNDNFGRGGNFSGRGGFGGSR<br>GGGGYGGSGDGYNGFGNDGSNFGGGGSYNDFGNYNNQSSNFGPMKGGNF<br>GGRSSGGSGGGGQYFAKPRNQGGYGGSSSSSSYGSGRRF
A1-LCD +NLS*	GSMASASSSQRGRSGSGNFGGGRGGGFGGNDNFGRGGNFSGRGGFGGSR<br>GGGGYGGSGDGYNGFGNDGSNFGGGGSYNDFGNYNNQSSNFGPMKGGNF<br>GGRSSGPYGGGGQYFAKPRNQGGYGGSSSSSSYGSGRRF
-12F+12Y*	GSMASASSSQRGRSGSGNYGGGRGGGYGGNDNYGRGGNYSGRGGYGGSR<br>GGGGYGGSGDGYNGYGNDGSNYGGGGSYNDYGNYNNQSSNYGPMKGGNY<br>GGRSSGGSGGGGQYYAKPRNQGGYGGSSSSSSYGSGRRY
+7F-7Y*	GSMASASSSQRGRSGSGNFGGGRGGGFGGNDNFGRGGNFSGRGGFGGSR<br>GGGGFGGSGDGFNGFGNDGSNFGGGGSFNDFGNFNNQSSNFGPMKGGNF<br>GGRSSGGSGGGGQFFAKPRNQGGFGGSSSSSSFGSGRRF
-4F-2Y*	GSMASASSSQRGRSGSGNSGGGRGGGFGGNDNFGRGGNSSGRGGFGGSR<br>GGGGYGGSGDGYNGFGNDGSNSGGGGSSNDFGNYNNQSSNFGPMKGGNF<br>GGRSSGGSGGGGQYSAKPRNQGGYGGSSSSSSSGSGRRF
-9F+6Y*	GSMASASSSQRGRSGSGNFGGGRGGGYGGNDNYGRGGNYSGRGGFGGSR<br>GGGGYGGSGDGYNGGGNDGSNYGGGGSYNDSGNYNNQSSNFGPMKGGNY<br>GGRSSGGSGGGGQYGAKPRNQGGYGGSSSSSSYGSGRRY
-8F+4Y*	GSMASASSSQRGRSGSGNFGGGRGGGYGGNDNGGRGGNYSGRGGFGGSR<br>GGGGYGGSGDGYNGGGNDGSNYGGGGSYNDSGNYNNQSSNFGPMKGGNY<br>GGRSSGGSGGGGQYGAKPRNQGGYGGSSSSSSYGSGRRF
-9F+3Y*	GSMASASSSQRGRSGSGNFGGGRGGGYGGNDNGGRGGNYSGRGGFGGSR<br>GGGGYGGSGDGYNGGGNDGSNYGGGGSYNDSGNGNNQSSNFGPMKGGNY<br>GGRSSGGSGGGGQYGAKPRNQGGYGGSSSSSSYGSGRRS
+12D*	GSMASADSSQRDRDDSGNFGDGRGGGFGGNDNFGRGGNFSDRGGFGGSR<br>GDGGYGGDGDGYNGFGNDGSNFGGGGSYNDFGNYNNQSSNFDPMKGGNF<br>GDRSSGPYDGGGQYFAKPRNQGGYGGSSSSSSYGSDRRF
+8D*	GSMASASSSQRDRSGSGNFGGGRDGGFGGNDNFGRGDNFSGRGDFGGSR<br>DGGGYGGSGDGYNGFGNDGSNFGGGGSYNDFGNYNNQSSNFGPMKGGNF<br>GGRSSDPYGGGGQYFAKPRNQDGYGGSSSSSSYDSGRRF
+4D*	GSMASASSSQRDRSGSGNFGGGRGGGFGGNDNFGRGGNFSGRGDFGGSR<br>GGGGYGGSGDGYNGFGNDGSNFGGGGSYNDFGNYNNQSSNFGPMKGGNF<br>GGRSSDPYGGGGQYFAKPRNQGGYGGSSSSSSYDSGRRF
-4D*	GSMASASSSQRGRSGSGNFGGGRGGGFGGNGNFGRGGNFSGRGGFGGSR<br>GGGGYGGSGGGYNGFGNSGSNFGGGGSYNGFGNYNNQSSNFGPMKGGNF<br>GGRSSGPYGGGGQYFAKPRNQGGYGGSSSSSSYGSGRRF
+2R*	GSMASASSSQRGRSGSGNFGGGRGGGFGGNDNFGRGGNFSGRGGFGGSR<br>GGGGYGGSGDGYNGFRNDGSNFGGGRYNDFGNYNNQSSNFGPMKGGNF<br>GGRSSGPYGGGGQYFAKPRNQGGYGGSSSSSSYGSGRRF

-6R*	GSMASASSSQGGRSGSGNFGGGRGGGFGGNDNFGGGGNFSGSGGFGGSR<br>GGGGYGGSGDGYNGFGNDGSNFGGGGSYNDFGNYNNQSSNFGPMKGGNF<br>GGSSSGPYGGGGQYFAKPGNQGGYGGSSSSSSYGSGGRF
-10R*	GSMASASSSQGGSSGSGNFGGGGGGGFGGNDNFGGGGNFSGSGGFGGSG<br>GGGGYGGSGDGYNGFGNDGSNFGGGGSYNDFGNYNNQSSNFGPMKGGNF<br>GGSSSGPYGGGGQYFAKPGNQGGYGGSSSSSSYGSGGGF
-3R+3K*	GSMASASSSQRGKSGSGNFGGGRGGGFGGNDNFGRGGNFSGRGGFGGSK<br>GGGGYGGSGDGYNGFGNDGSNFGGGGSYNDFGNYNNQSSNFGPMKGGNF<br>GGRSSGGSGGGGQYFAKPRNQGGYGGSSSSSSYGSGRKF
-6R+6K*	GSMASASSSQKGKSGSGNFGGGRGGGFGGNDNFGKGGNFSGRGGFGGSK<br>GGGGYGGSGDGYNGFGNDGSNFGGGGSYNDFGNYNNQSSNFGPMKGGNF<br>GGKSSGGSGGGGQYFAKPRNQGGYGGSSSSSSYGSGRKF
+7K+12D*	GSMASADSSQRDRDDKGNFGDGRGGGFGGNDNFGRGGNFSDRGGFGGSR<br>GDGKYGGDGDKYNGFGNDGKNFGGGGSYNDFGNYNNQSSNFDPMKGGNF<br>KDRSSGPYDKGGQYFAKPRNQGGYGGSSSSKSYGSDRRF
-20G+20S**	GSMASASSSQRSRSGSGNFSGSRSGSFSGNDNFGRSGNFSGRSGFGGSR<br>SGGGYSGSGDSYNSFGNDGSNFSGSGSYNDFGNYNNQSSNFGPMKSGNF<br>GGRSSGSSGGSGQYFAKPRNQGSYSGSSSSSSYGSSRRF
-30G+30S**	GSMASASSSQRSRSSSGNFSGSRSGSFSGNDNFGRSGNFSGRSGFSGSR<br>SGSGYSGSSDSYNSFGNDSSNFSGSSSYNDFGNYNNQSSNFGPMKSGNF<br>SGRSSSSSGSSGQYFAKPRNQGSYSGSSSSSSYSSSRRF
-12F+12Y-<br>20G+20S**	GSMASASSSQRSRSGSGNYSGSRSGSYSGNDNYGRSGNYSGRSGYGGSR<br>SGGGYSGSGDSYNSYGNDGSNYSGSGSYNDYGNYNNQSSNYGPMKSGNY<br>GGRSSGSSGGSGQYYAKPRNQGSYSGSSSSSSYGSSRRY
-12F+12Y-<br>30G+30S**	GSMASASSSQRSRSSSGNYSGSRSGSYSGNDNYGRSGNYSGRSGYSGSR<br>SGSGYSGSSDSYNSYGNDSSNYSGSSSYNDYGNYNNQSSNYGPMKSGNY<br>SGRSSSSSGSSGQYYAKPRNQGSYSGSSSSSSYSSSRRY
+7F-7Y-<br>20G+20S**	GSMASASSSQRSRSGSGNFSGSRSGSFSGNDNFGRSGNFSGRSGFGGSR<br>SGGGFSGSGDSFNSFGNDGSNFSGSGSFNDFGNFNNQSSNFGPMKSGNF<br>GGRSSGSSGGSGQFFAKPRNQGSFSGSSSSSSFGSSRRF
+7F-7Y -<br>30G+30S**	GSMASASSSQRSRSSSGNFSGSRSGSFSGNDNFGRSGNFSGRSGFSGSR<br>SGSGFSGSSDSFNSFGNDSSNFSGSSSFNDFGNFNNQSSNFGPMKSGNF<br>SGRSSSSSGSSGQFFAKPRNQGSFSGSSSSSSFSSSRRF
-23S+23T**	GSMATATTTQRGRTGTGNFGGGRGGGFGGNDNFGRGGNFTGRGGFGGTR<br>GGGGYGGTGDGYNGFGNDGTNFGGGGTYNDFGNYNNQTTNFGPMKGGNF<br>GGRTTGGTGGGGQYFAKPRNQGGYGGTTTTTTYGTGRRF
-14N+14Q**	GSMASASSSQRGRSGSGQFGGGRGGGFGGQDQFGRGGQFSGRGGFGGSR<br>GGGGYGGSGDGYQGFGQDGSQFGGGGSYQDFGQYQQQSSQFGPMKGGQF<br>GGRSSGGSGGGGQYFAKPRQQGGYGGSSSSSSYGSGRRF
-14N-4Q+18G**	GSMASASSSGRGRSGSGGFGGGRGGGFGGGDGFGRGGGFSGRGGFGGSR<br>GGGGYGGSGDGYGGFGGDGSGFGGGGSYGDFGGYGGGSSGFGPMKGGGF<br>GGRSSGGSGGGGGYFAKPRGGGGYGGSSSSSSYGSGRRF
+7R+10D	GSMASADSSQRDRDGRGNFGDGRGGGFGGNDNFGRGGNFSDRGGFGGSR<br>GGGRYGGDGDRYNGFGNDGRNFGGGGSYNDFGNYNNQSSNFDPMKGGNF<br>RDRSSGPYDRGGQYFAKPRNQGGYGGSSSSRSYGSDRRF

+7R+12D	GSMASADSSQRDRDDRGNFGDGRGGGFGGNDNFGRGGNFSDRGGFGGSR<br>GDGRYGGDGDRYNGFGNDGRNFGGGGSYNDFGNYNNQSSNFDPMKGGNF<br>RDRSSGPYDRGGQYFAKPRNQGGYGGSSSSRSYGSDRRF
-10F +7R+12D	GSMASADSSQRDRDDRGNFGDGRGGGGGGNDNFGRGGNGSDRGGGGGSR<br>GDGRYGGDGDRYNGGGNDGRNGGGGGSYNDGGNYNNQSSNGDPMKGGNG<br>RDRSSGPYDRGGQYGAKPRNQGGYGGSSSSRSYGSDRRG
-12F+12Y-10R	GSMASASSSQGGSSGSGNYGGGGGGGYGGNDNYGGGGNYSGSGGYGGSG<br>GGGGYGGSGDGYNGYGNDGSNYGGGGSYNDYGNYNNQSSNYGPMKGGNY<br>GGSSSGPYGGGGQYYAKPGNQGGYGGSSSSSSYGSGGGY
FUS-LCD	GSMASNDYTQQATQSYGAYPTQPGQGYSQQSSQPYGQQSYSGYSQSTDTSG<br>YGQSSYSSYGQSQNTGYGTQSTPQGYGSTGGYGSSQSSQSSYGQQSSYPGYG<br>QQPAPSSTSGSYGSSSQSSSYGQPQSGSYSQQPSYGGQQQSYGQQQSYNPPQ<br>GYGQQNQYNSSSGGGGGGGGGGNYGQDQSSMSSGGGSGGGYGNQDQSGG<br>GGSGGYGQQDRG
DDX4-IDR	MGDEDWEAEINPHMSSYVPIFEKDRYSGENGDNFNRTPASSSEMDDGPSRR<br>DHFMKSGFASGRNFGNRDAGECNKRDNTSTMGGFGVGKSFGNRGFSNSRF<br>EDGDSSGFWRESSNDCEDNPTRNRGFSKRGGYRDGNNSEASGPYRRGGRG<br>SFRGCRGGFGLGSPNNDLDPDECMQRTGGLFGSRRPVLSGTGNGDTSQSRS<br>GSGSERGGYKGLNEEVITGSGKNSWKSEAEGGES

Supplementary Table 3: Initial parameterization of sticker-and-spacer model using GPBO

Pairwise Interaction	Lower Bound	Upper Bound	Final Value
Tyr-Tyr	-32	-15	-19.3
Tyr-Phe	-30	-12	-15.2
Phe-Phe	-25	-5	-13.4
Arg-Aro	-25	-8	-11.0
Lys-X	-3	3	-0.389
X-X	-4	-2	-2.95

Supplementary Table 4: Final parameterization of sticker-and-spacer model

Pairwise Interaction	Final Value
Tyr-Tyr	-19.3
Tyr-Phe	-15.2
Phe-Phe	-13.4
Arg-Aro	-11.0
Lys-X	-0.389
Thr-X	-2.35
Ser-X	-2.65
Gln-X	-2.75
Asn-X	-3.05
Gly-X	-3.15
X-X	-2.95

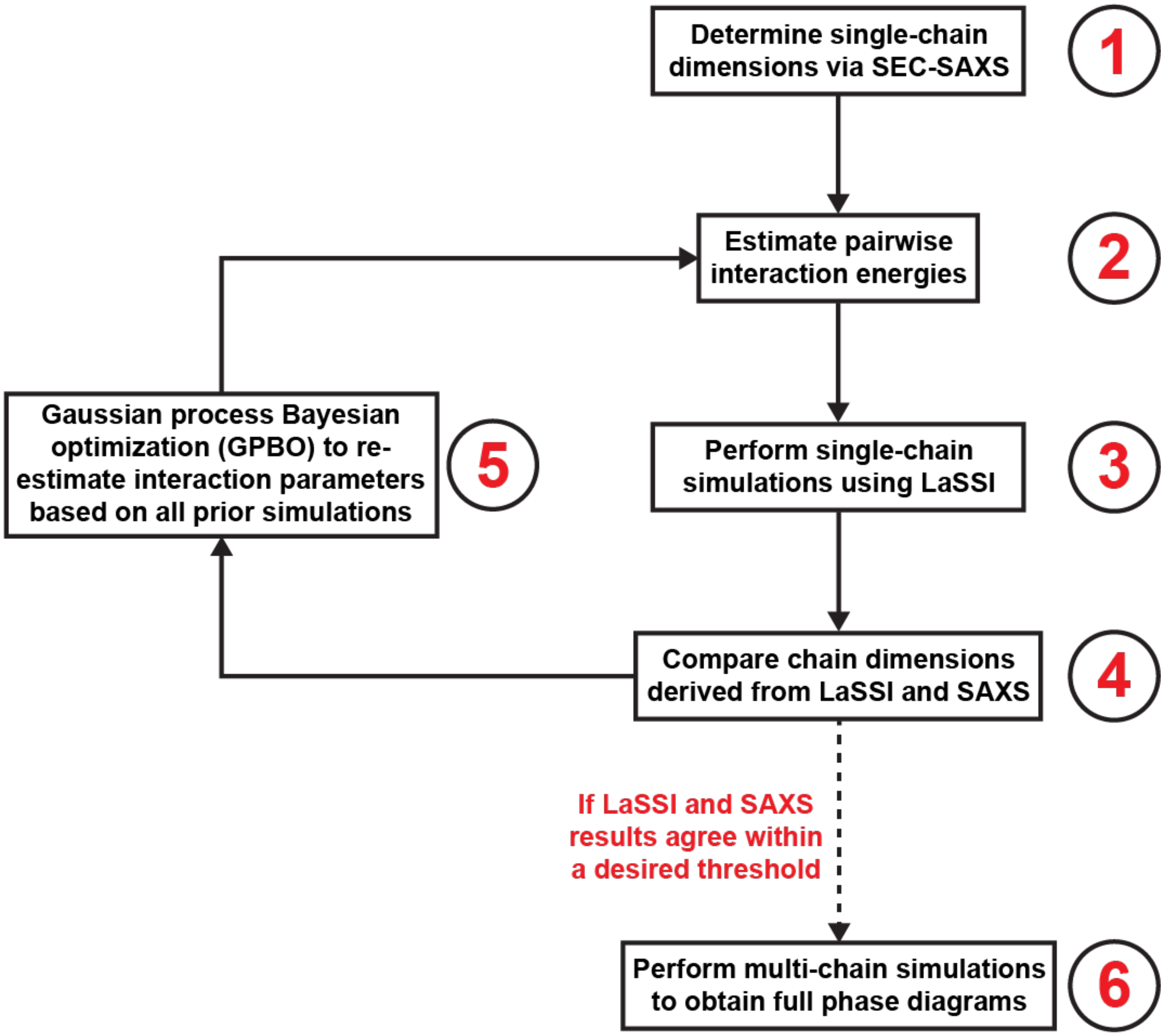

Supplementary Figure 1: Flowchart describing the Gaussian process Bayesian optimization[7] used to parameterize the computational model.

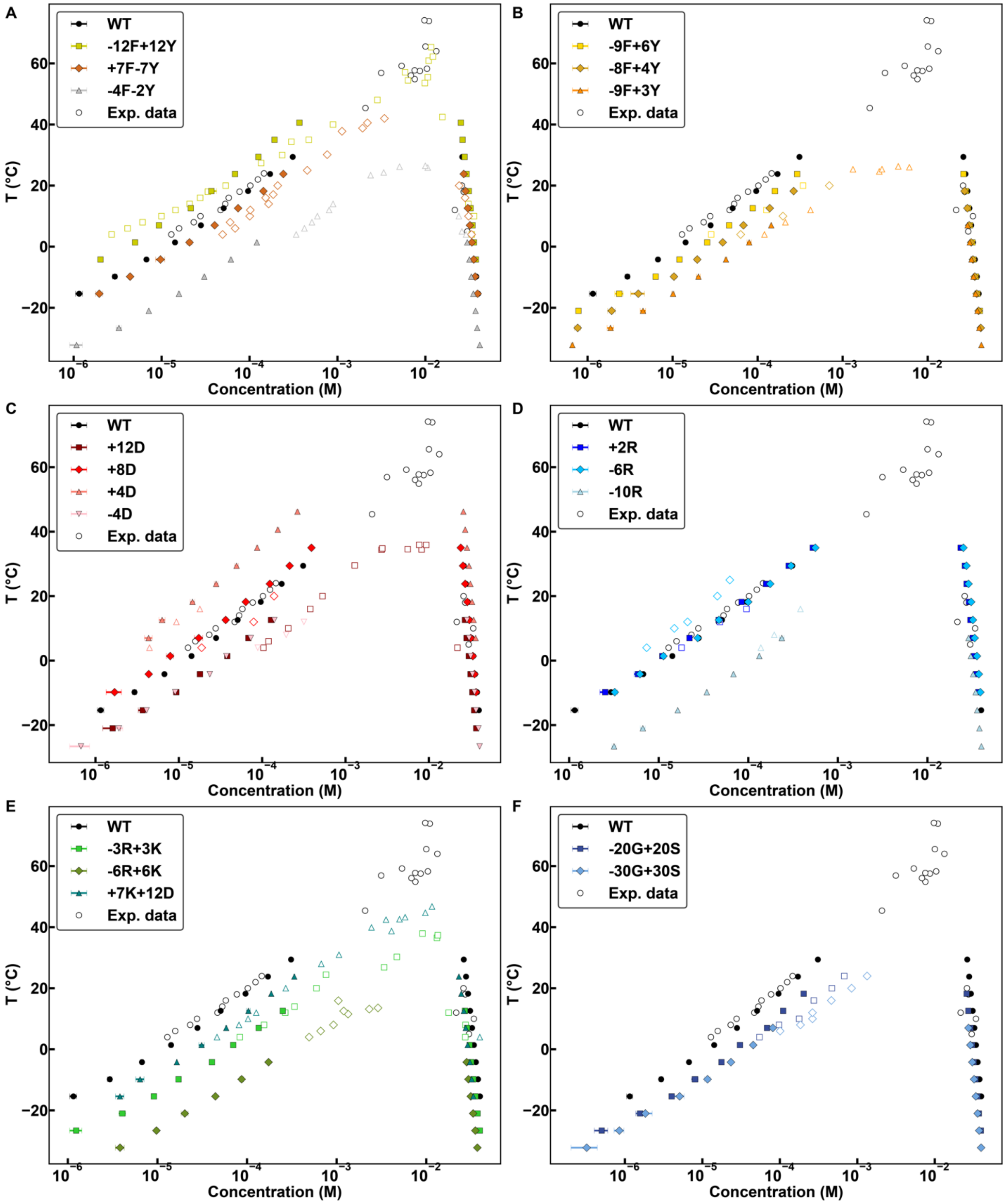
A
WT
-12F+12Y
+7F-7Y
-4F-2Y
Exp. data
B
WT
-9F+6Y
-8F+4Y
-9F+3Y
Exp. data
C
WT
+12D
+8D
+4D
-4D
Exp. data
D
WT
+2R
-6R
-10R
Exp. data
E
WT
-3R+3K
-6R+6K
+7K+12D
Exp. data
F
WT
-20G+20S
-30G+30S
Exp. data
T (°C)
Concentration (M)

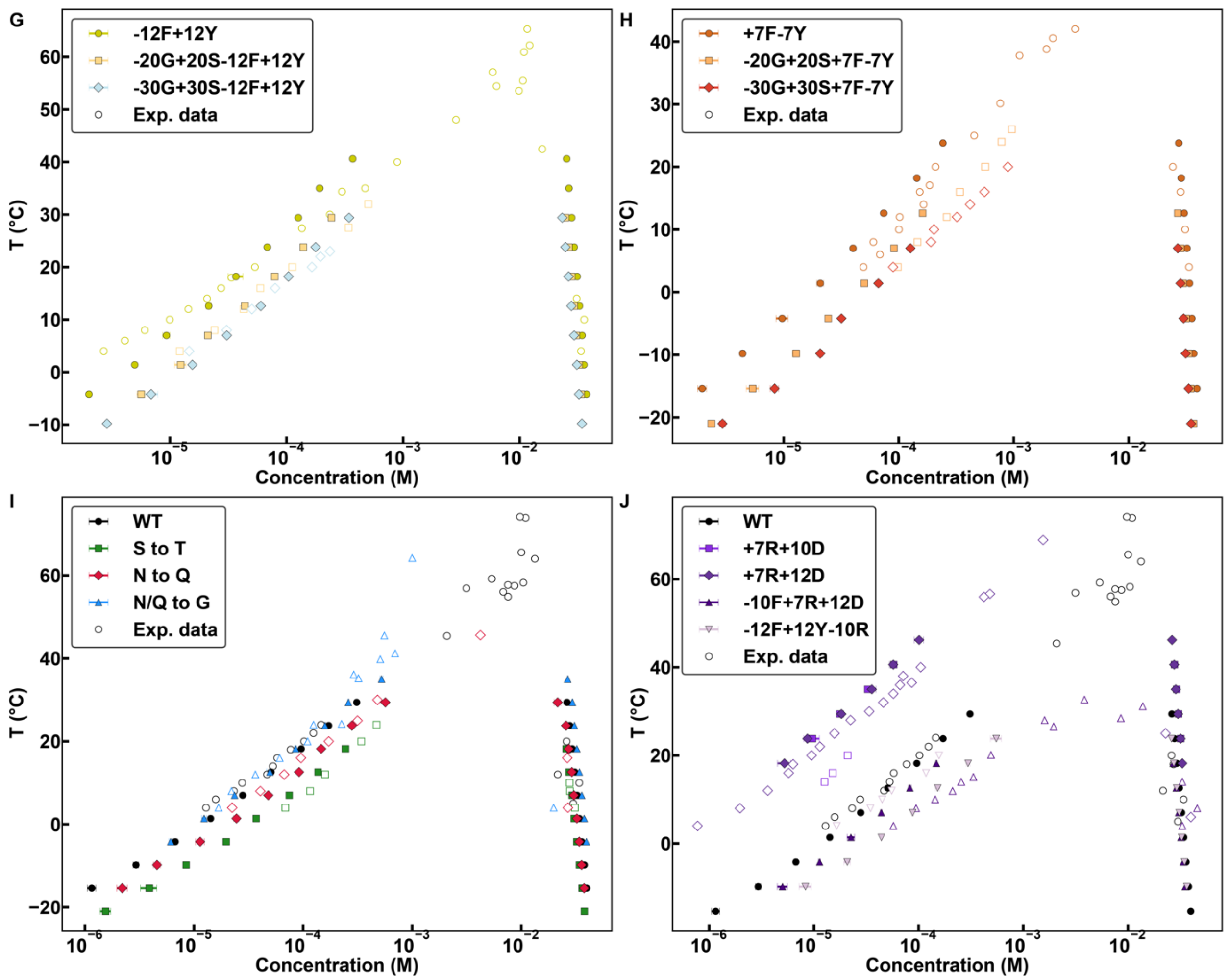

Supplementary Figure 2: Calculated binodals (solid markers) of all A1-LCD variants used in this study plotted alongside binodals derived from experiments (open markers). Temperature and concentration are converted from simulation units to experimental units by the same scaling factors for each variant (5.6 and 0.6, respectively). In most cases, the WT A1-LCD is shown for reference. -12F+12Y and +7F-7Y are shown for reference in **G** and **H**, respectively. Variants depicted in **A-E** were used in the Gaussian process Bayesian optimization. Variants depicted in **F-I** were used to manually parameterize spacer interaction energies. Variants depicted in **J** (excluding WT) were not used to parameterize the computational model. Error bars indicate standard errors from the mean across 3 replicates. Experimental data are from Bremer et al., [3]. Source data are provided as a Source Data file.

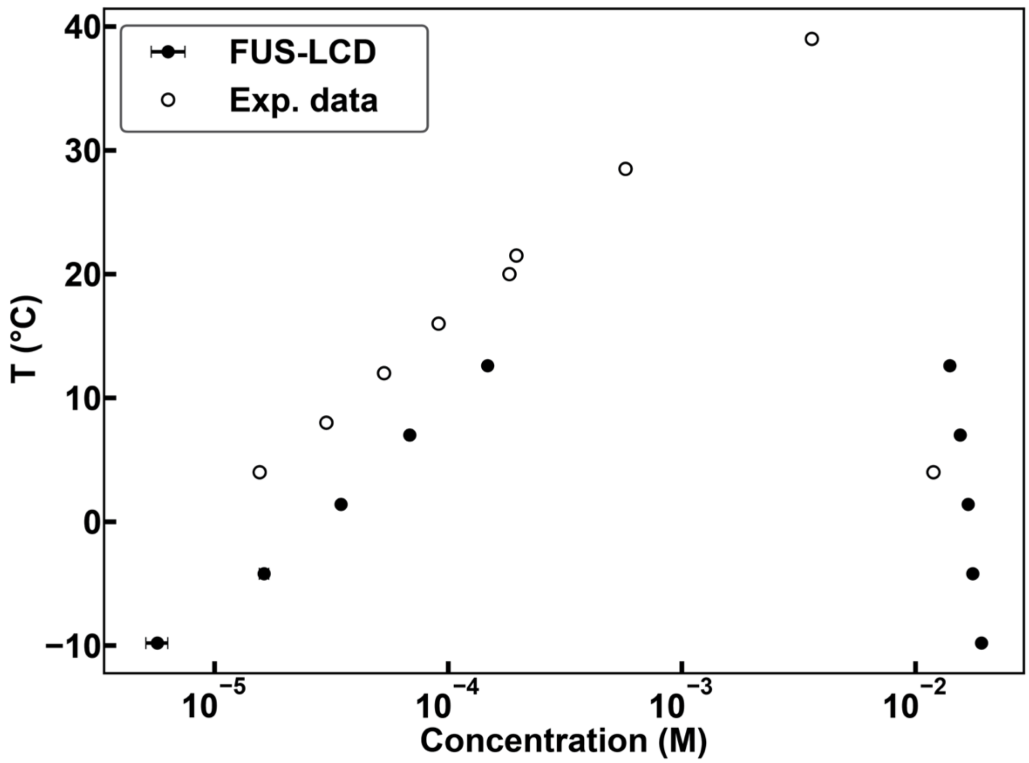

Supplementary Figure 3: Calculated phase diagram (solid markers) and experimental phase diagram (open markers) of the FUS-LCD. Temperature and concentration were converted from simulation units to experimental units *viz*., degree-Celsius and moles per liter using the scaling factors introduced by Martin et al., for the A1-LCD variants (5.6 and 0.6, respectively. The deviation from the experimental data is partly due to the difference in the lengths of the A1-LCD and FUS-LCD, with the latter being ~1.7 times longer. Therefore, using the same scaling factor causes an underestimation of the transition temperature for the FUS-LCD for each of the concentrations. However, using a scaling factor of 5.7 instead of 5.6 leads to a better alignment of the measured and computed binodals. We do not choose this route for presenting our results or quantifying the EMRSL. As a result, the EMRSL that we compute is in fact an overestimate of the inaccuracy of the computed binodal for FUS-LCD as referenced against the binodal inferred from experiments. Error bars, which are included but invisible due to their small size, indicate standard errors from the mean across 3 replicates. Source data are provided as a Source Data file.

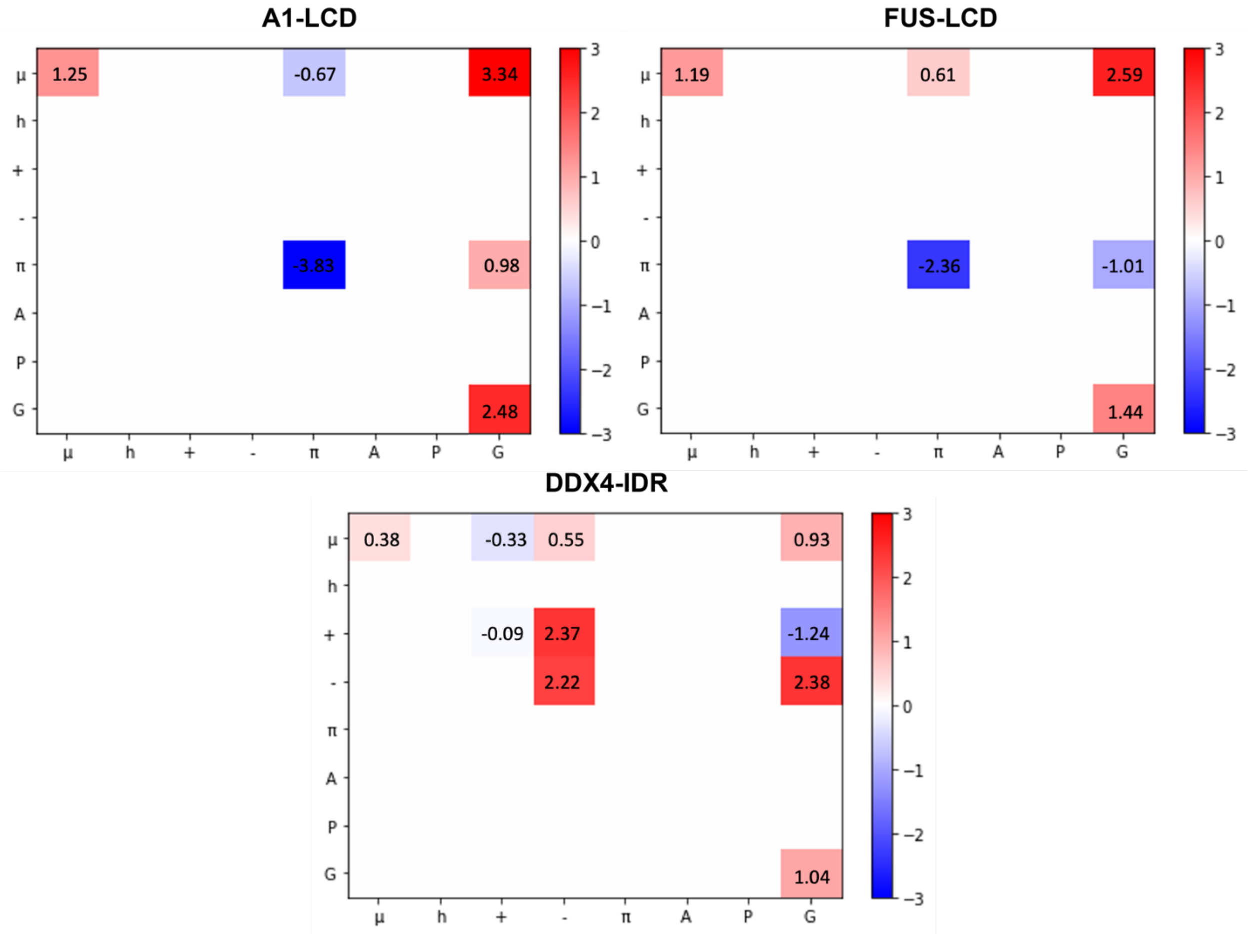

Supplementary Figure 4: NARDINI analysis of various IDRs. Positive z-scores indicate non-random segregation between two types of residues or a blocky distribution of one type of residue. Negative z-scores indicate non-random mixing between two types of residues or a uniform distribution of one type of residue. Z-scores close to 0 indicate random patterning. Only the upper half of the matrix, as well as the diagonal, are filled in, to avoid redundancy. In the case where a square is unlabeled, the IDR is composed of less than 10% of one or both residue categories of interest. In this case, the residue type is too depleted to appropriately quantify the non-randomness of its patterning with other residue types. Types of residues are categorized as follows: polar (μ): (Ser, Thr, Asn, Gln, Cys, His), hydrophobic (h): (Iso, Leu, Met, Val), positive (+): (Arg, Lys), negative (-): (Glu, Asp), aromatic (π): (Phe, Tyr, Trp), alanine (A), proline, (P), and glycine (G).

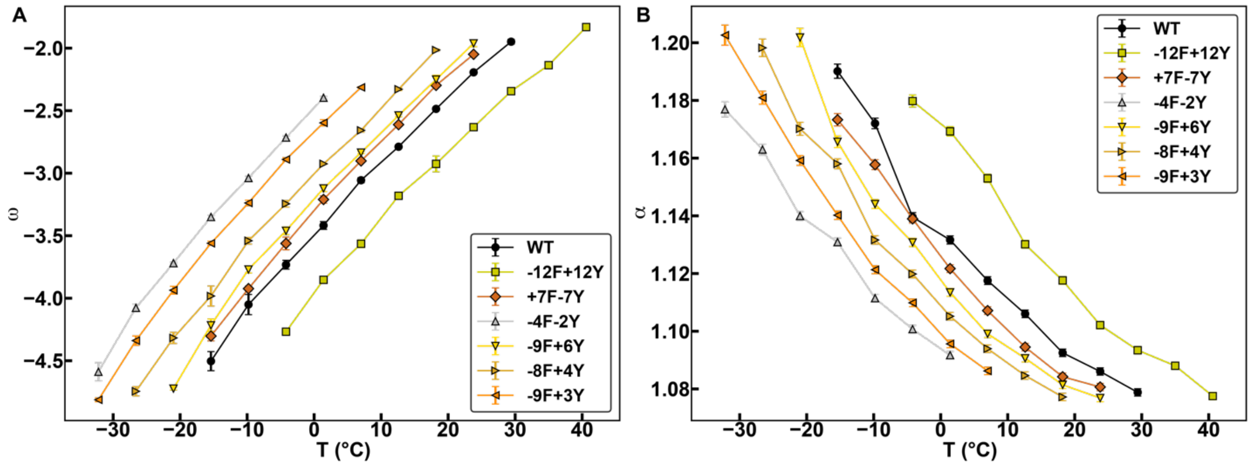

Supplementary Figure 5: Swelling ratios and widths of two-phase regimes for different variants. (A) Average width of the two-phase regime, ω, plotted against temperature for aromatic variants of A1-LCD. **(B)** Average swelling ratio, α, plotted against temperature for aromatic variants of A1-LCD. Error bars indicate standard errors from the mean across 3 replicates. Source data are provided as a Source Data file.

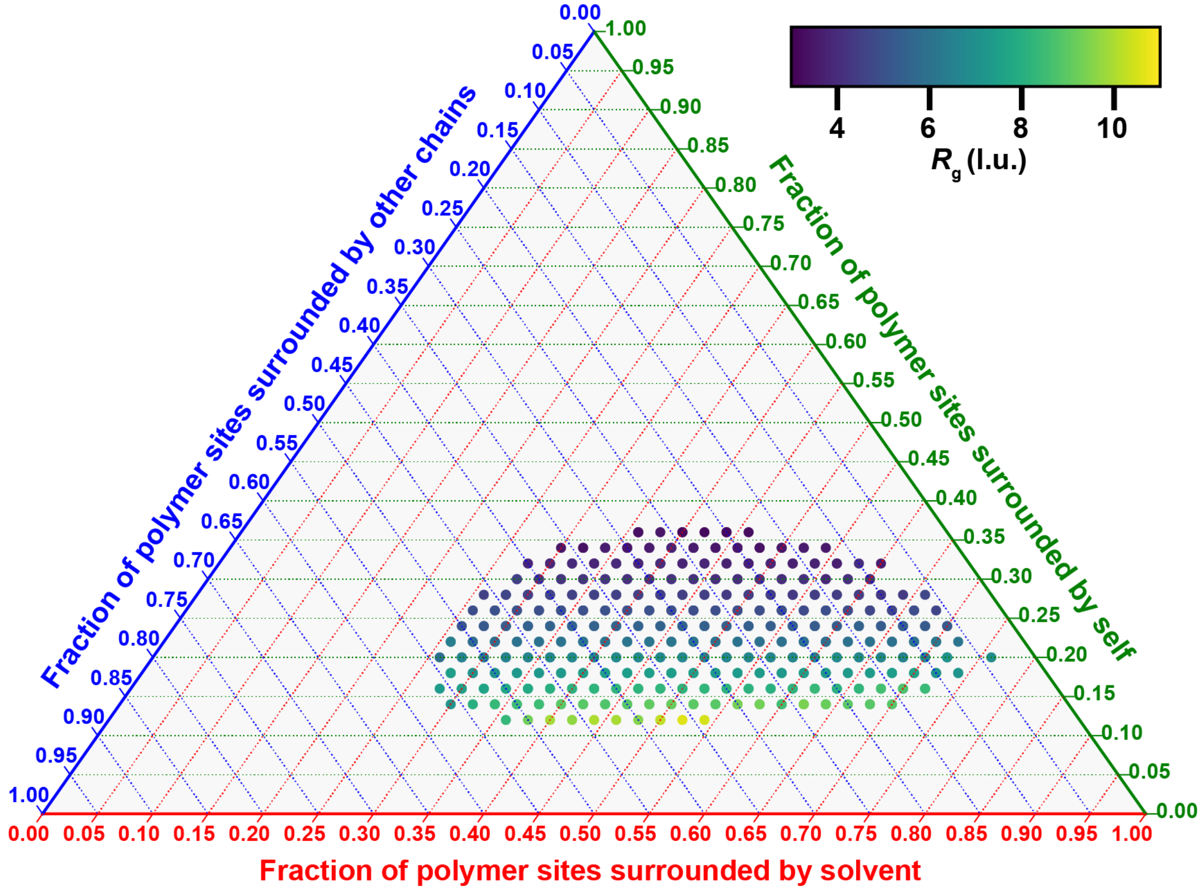

Supplementary Figure 6: Ternary plot depicting the average radius of gyration (as represented by the color bar) of a chain in the condensate as a function of the fraction of its polymer sites that are surrounded by solvent (lower axis; red), by other chains (upper left axis; blue), or by itself (upper right axis; green). Results are shown here for the wild-type A1-LCD at ω = -4.5. The direction of the tick marks along each axis is the direction to follow for that axis. l.u. refers to lattice units. Source data are provided as a Source Data file.

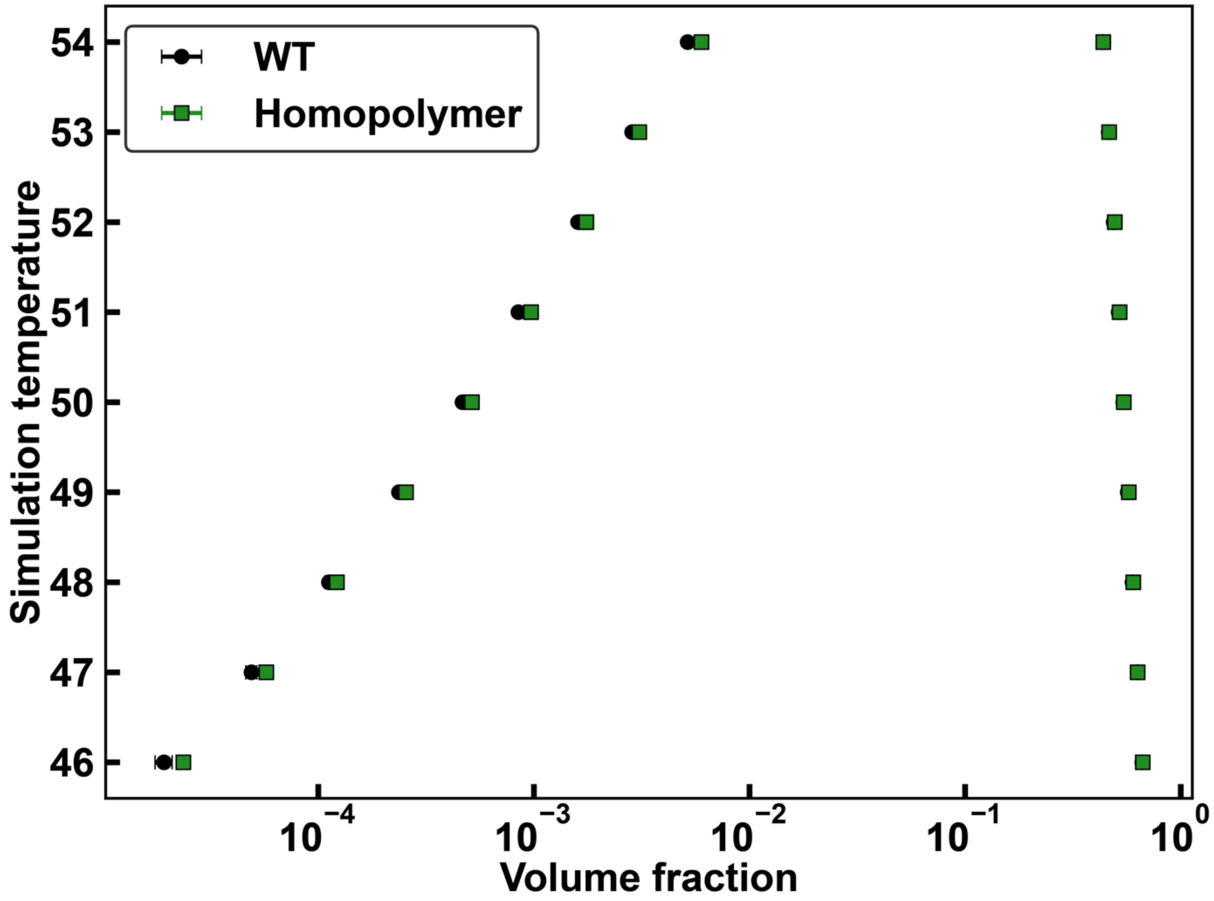

Supplementary Figure 7: Calculated binodals of the wild-type A1-LCD and an equivalent homopolymer whose pairwise interaction energies are all set to -3.3. Error bars indicate standard errors from the mean across 3 replicates. Source data are provided as a Source Data file.

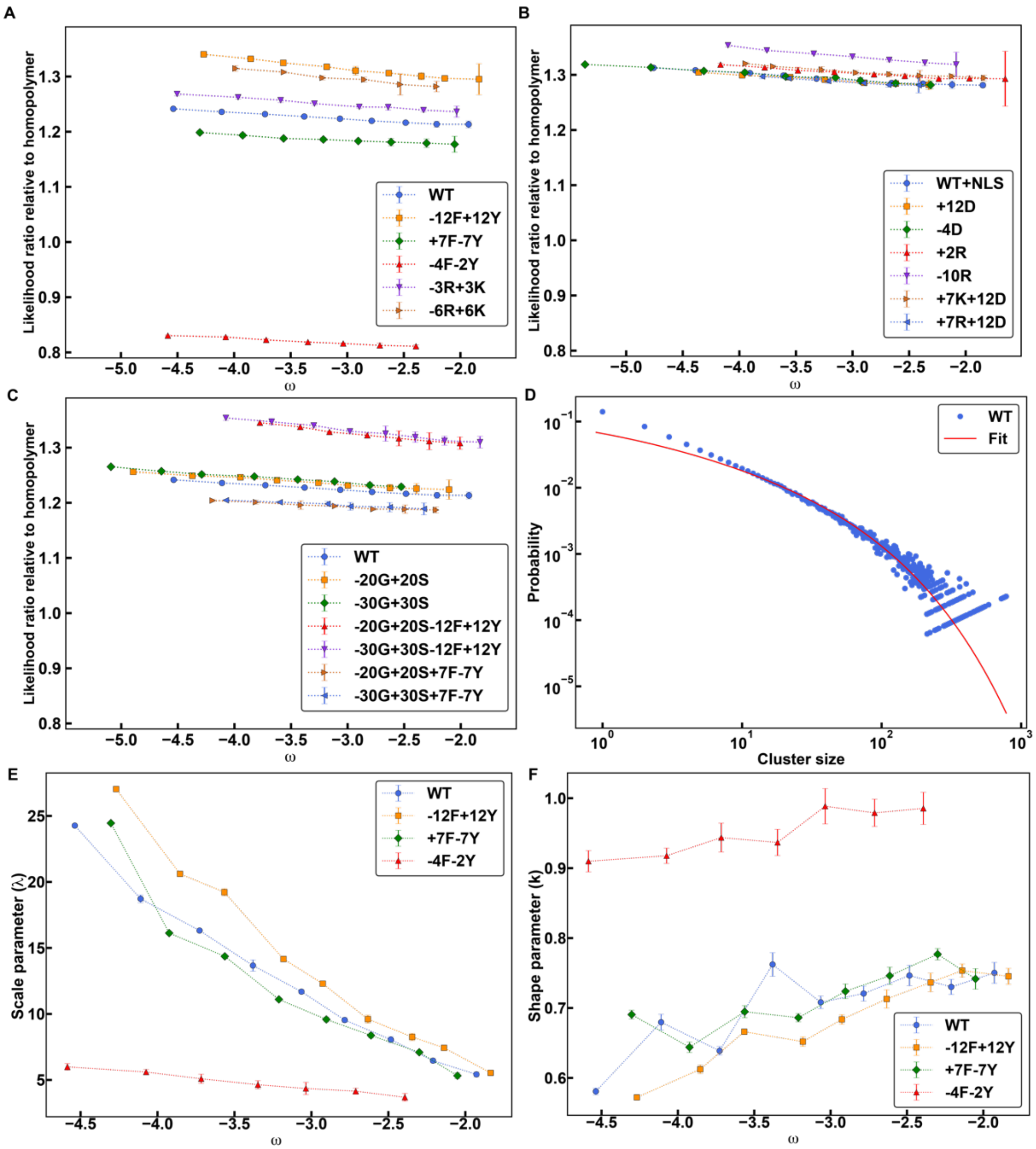

Supplementary Figure 8: (A-C) Likelihood ratio that a sticker residue (Tyr or Phe) within a simulated condensate forms a crosslink with another sticker versus a spacer for variants of A1-LCD. This parameter is normalized by the likelihood ratio for the homopolymer, assuming the same sticker-spacer architecture as the wild-type A1-LCD. Larger values suggest that stickers within the condensate are more likely to be surrounded by other stickers. The parameter is plotted against the width of the two-phase regime, as defined in Fig. 2. The variants in **(B)** contain a nuclear localization signal (NLS), which replaces a GS motif with a PY motif. **(D)** A representative log-log plot of the probability for a sticker to be in a cluster of a particular size within the condensate for the wild-type A1-LCD at $\omega = -4.5$. The cluster size is defined as the number of stickers comprising the largest connected component. The solid red curve represents a fit to the data set assuming a discrete Weibull distribution past a certain cluster size. **(E-F)** The scale parameter, λ **(E),** and the shape parameter, k **(F),** for the fits in **(D)** at various temperatures. Error bars in **(A-C)** indicate standard errors from the mean across 3 replicates. Error bars in **(E-F)** indicate standard deviations about the mean across 3 replicates. Source data are provided as a Source Data file.

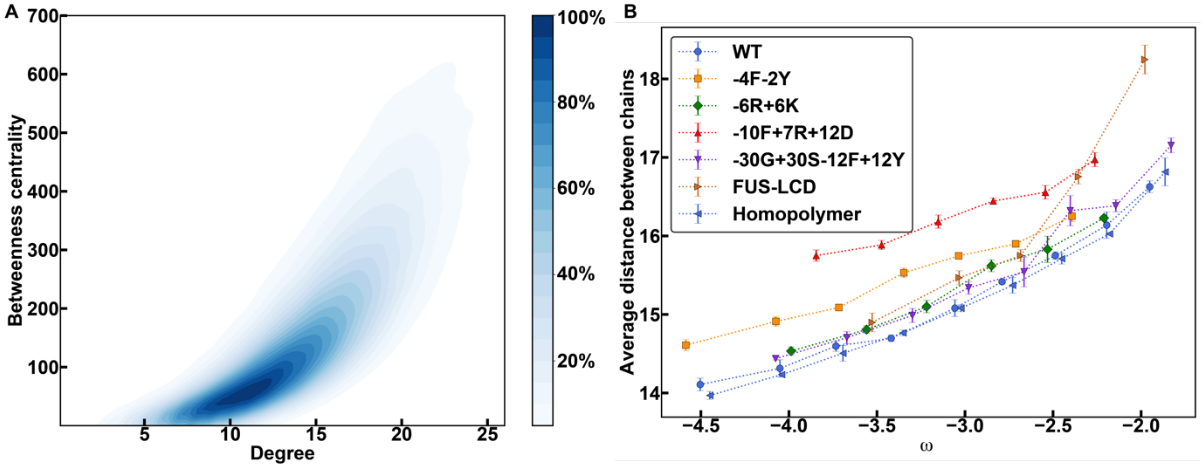

Supplementary Figure 9: (A) Bivariate distribution of degree and betweenness centrality for the WT A1-LCD at the lowest value of ω, -4.5. We see a strong positive correlation between the two parameters. Data in the top-right of the distribution indicate "hubs" that are particularly well-connected (high degree) and central. **(B)** The average pairwise distance between chains with the top 5% betweenness centralities at various ω values for a diverse set of 7 constructs. Error bars indicate standard errors from the mean across 3 replicates. Source data are provided as a Source Data file.

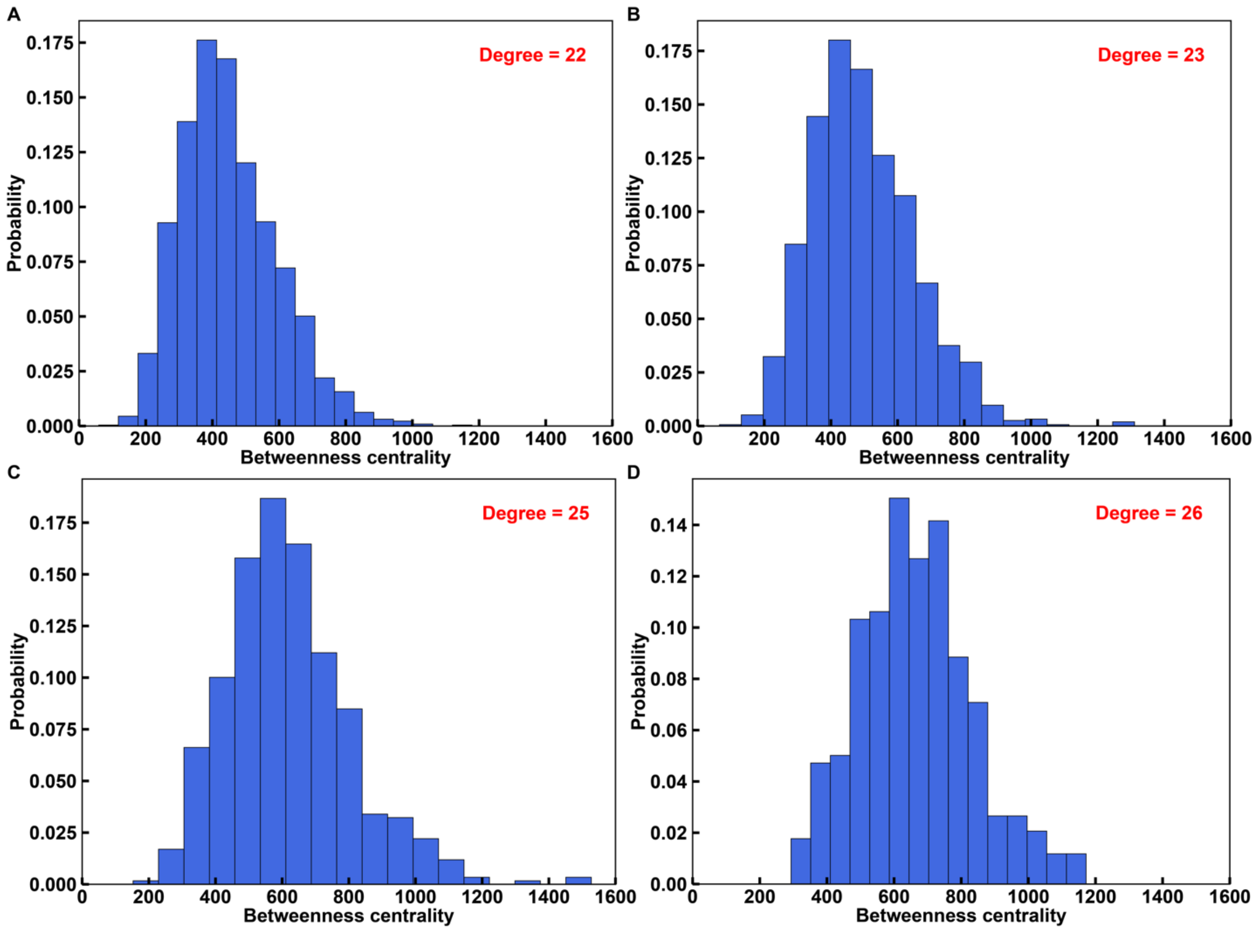

Supplementary Figure 10: Betweenness centrality distributions for chains with degree 22 (A), 23 (B), 25 (C), and 26 (D) at the smallest value of ω, -4.5, for the WT A1-LCD. We note that all distributions appear right skewed. The number of samples used in each panel are 2231 **(A)**, 1544 **(B)**, 589 **(C)**, and 339 **(D)**. Source data are provided as a Source Data file.

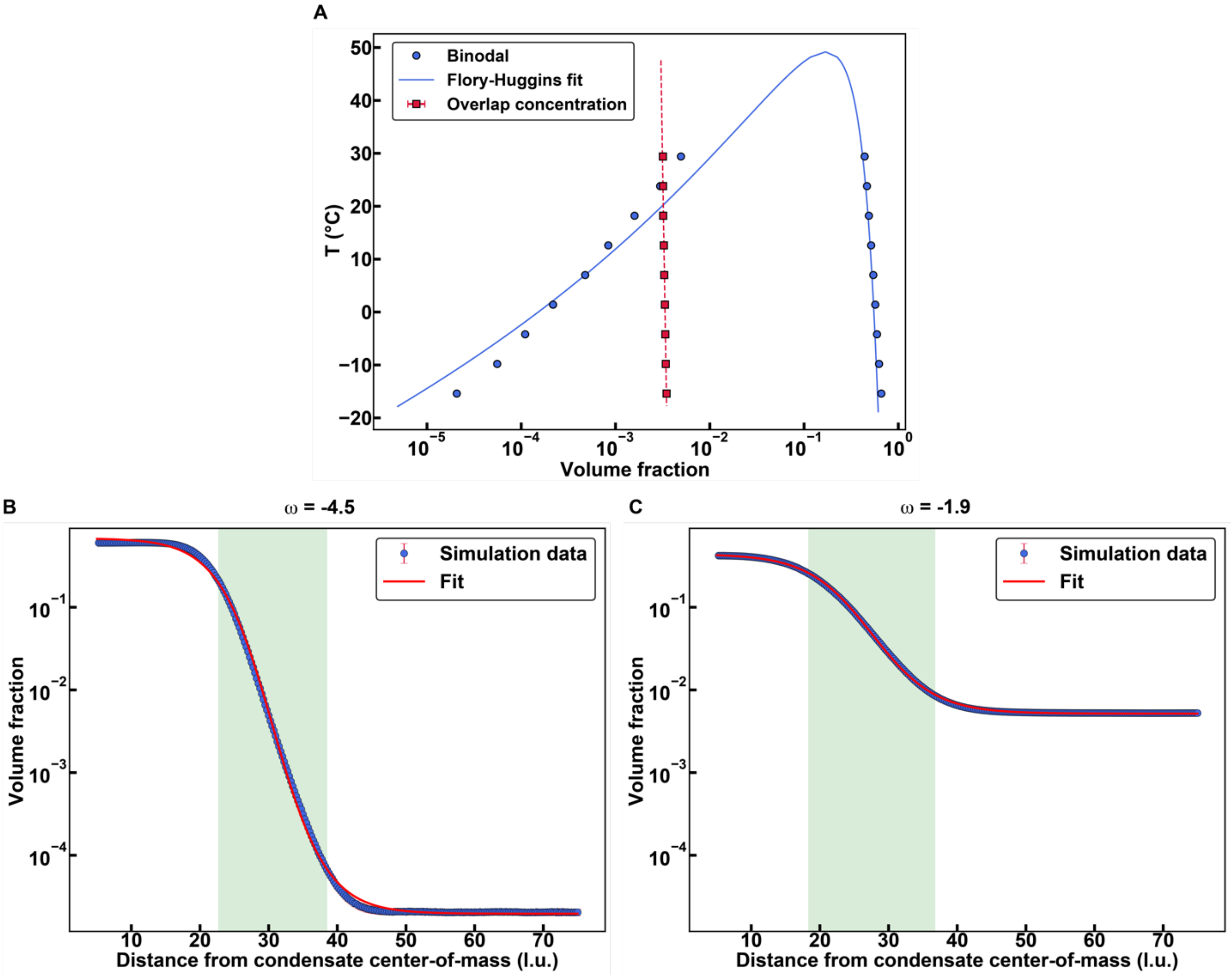

Supplementary Figure 11: (A) Calculated binodal (circular markers) and calculated overlap concentration (square markers) of the wild-type A1-LCD. The solid blue curve represents a fit using the Flory-Huggins model. The fit is made to the binodal for the equivalent homopolymer from Supplementary Figure 7. The dashed red line represents a linear fit of the homopolymer overlap concentration to guide the eye. **(B-C)** Radial density plots of simulations of the wild-type A1-LCD at two different ω values. The solid red curves correspond to fits of the data as described in the main text. The translucent green rectangles represent the interfacial regions determined by the respective fits. Error bars in **(A)** indicate standard errors from the mean across 3 replicates. Source data are provided as a Source Data file.

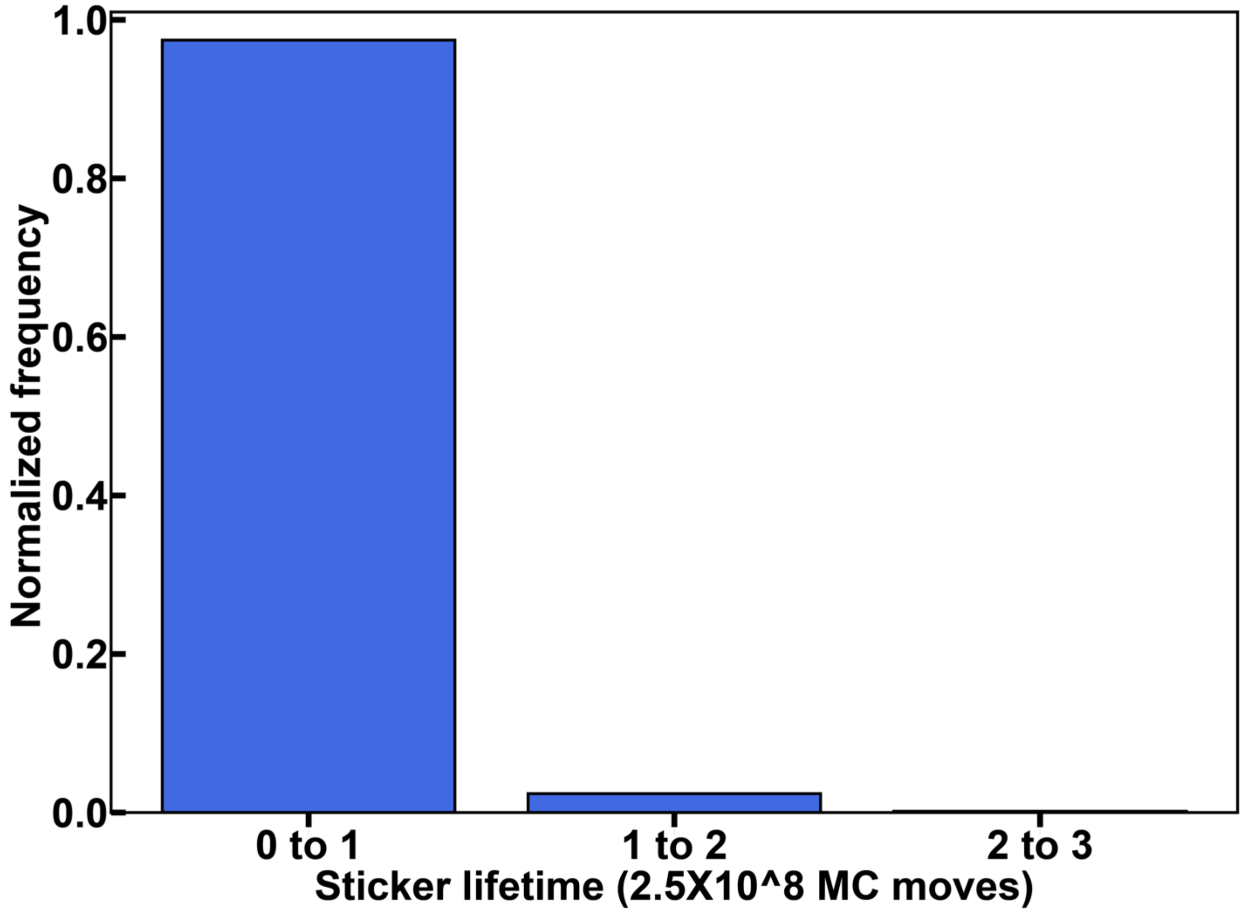

Supplementary Figure 12: Histogram of sticker lifetimes in dynamics simulations of WT A1-LCD at a simulation temperature of 46 (ω=-4.5). Source data are provided as a Source Data file.

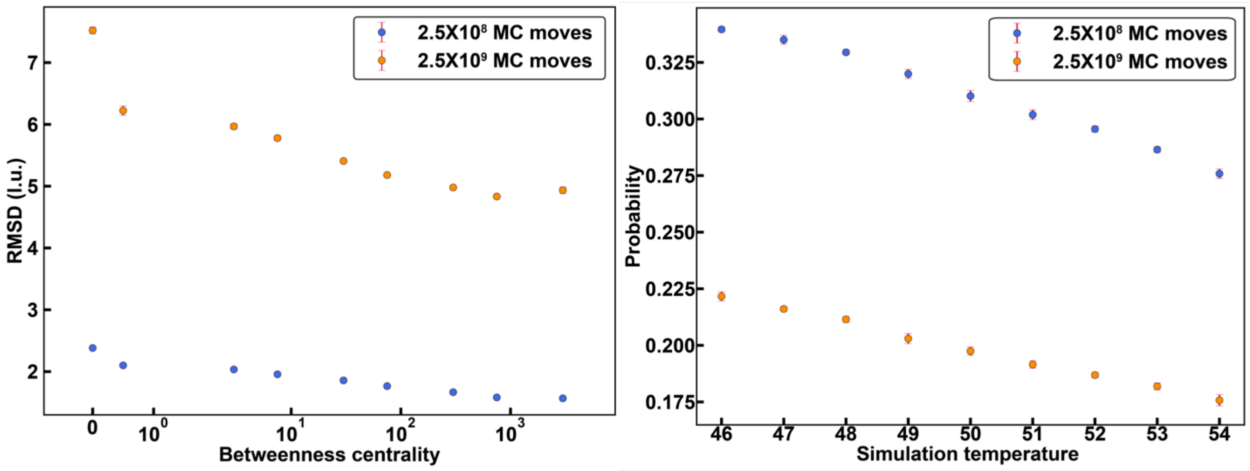

Supplementary Figure 13: Dynamics analyses of WT A1-LCD condensate simulations. (A) The root-mean-square-displacement (RMSD) of chains binned by their betweenness centrality at ω=-3.38. Betweenness centrality bins are as follows: 0, 0-1, 1-5, 5-10, 10-50, 50-100, 100-500, 500-1000, 1000+. **(B)** The probability that a chain that is one of the 5% most connected chains (based on its betweenness centrality) stays in the top 5% after the given number of MC moves plotted against simulation temperature. Both analyses are shown with time-steps of 2.5×10^8 Monte Carlo steps and 2.5×10^9 Monte Carlo steps. Error bars, although invisible due to their small size, represent standard errors from the mean across 5 replicates. Simulations were performed as described in the SM for dynamics simulations. Source data are provided as a Source Data file.

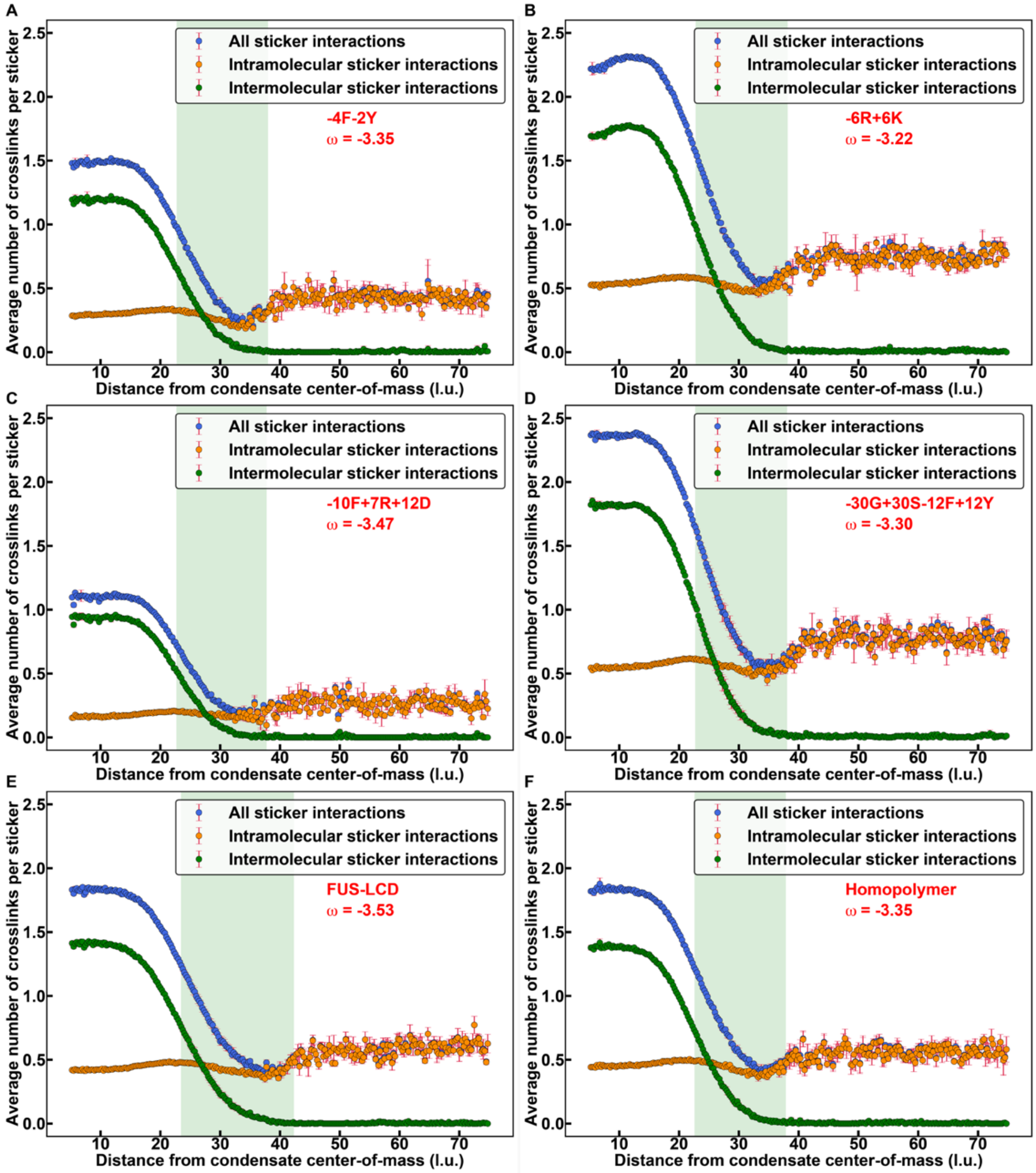

Supplementary Figure 14: Average number of sticker-sticker crosslinks per sticker plotted against the distance from the condensate center-of-mass for various protein constructs. (A) -4F-2Y at ω = -3.35, **(B)** -6R+6K at ω = -3.22, **(C)** -10F+7R+12D at ω = -3.47, **(D)** -30G+30S-12F+12Y at ω = -3.22, **(E)** -FUS-LCD at ω = -3.22, **(F)** homopolymer at ω = -3.22. Stickers signify Tyr or Phe residues. In the case of the homopolymer, which has equivalent interactions between every pair of residues, the chain is considered to have the same architecture as the WT A1-LCD. Representative simulations were chosen such that the value of ω was as close as possible to -3.38, the ω value of the WT analyses shown in Fig. 4-5. The translucent green boxes represent the interfacial region as determined by the logistic fit. In all panels, error bars signify standard errors about the mean across 3 replicates and l.u. is lattice units. Source data are provided as a Source Data file.

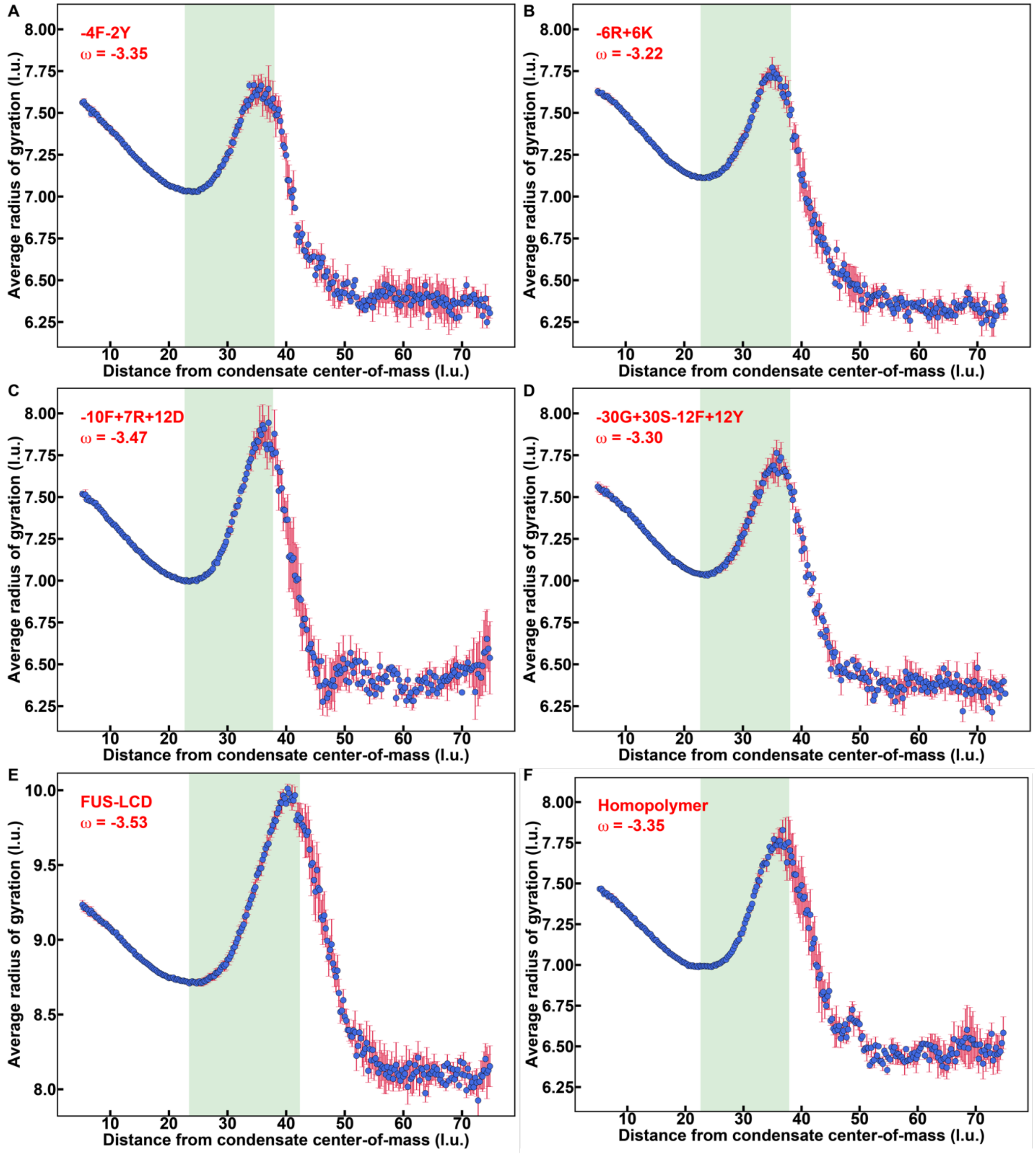

Supplementary Figure 15: Average R_g of a chain plotted against the distance from the condensate center-of-mass for various protein constructs. (A) -4F-2Y at ω = -3.35, **(B)** -6R+6K at ω = -3.22, **(C)** -10F+7R+12D at ω = -3.47, **(D)** -30G+30S-12F+12Y at ω = -3.22, **(E)** -FUS-LCD at ω = -3.22, **(F)** homopolymer at ω = -3.22. Representative simulations were chosen such that the value of ω was as close as possible to -3.38, the ω value of the WT analyses shown in Fig. 4-5. The translucent green boxes represent the interfacial region as determined by the logistic fit. In all panels, error bars signify standard errors about the mean across 3 replicates and l.u. is lattice units. Source data are provided as a Source Data file.

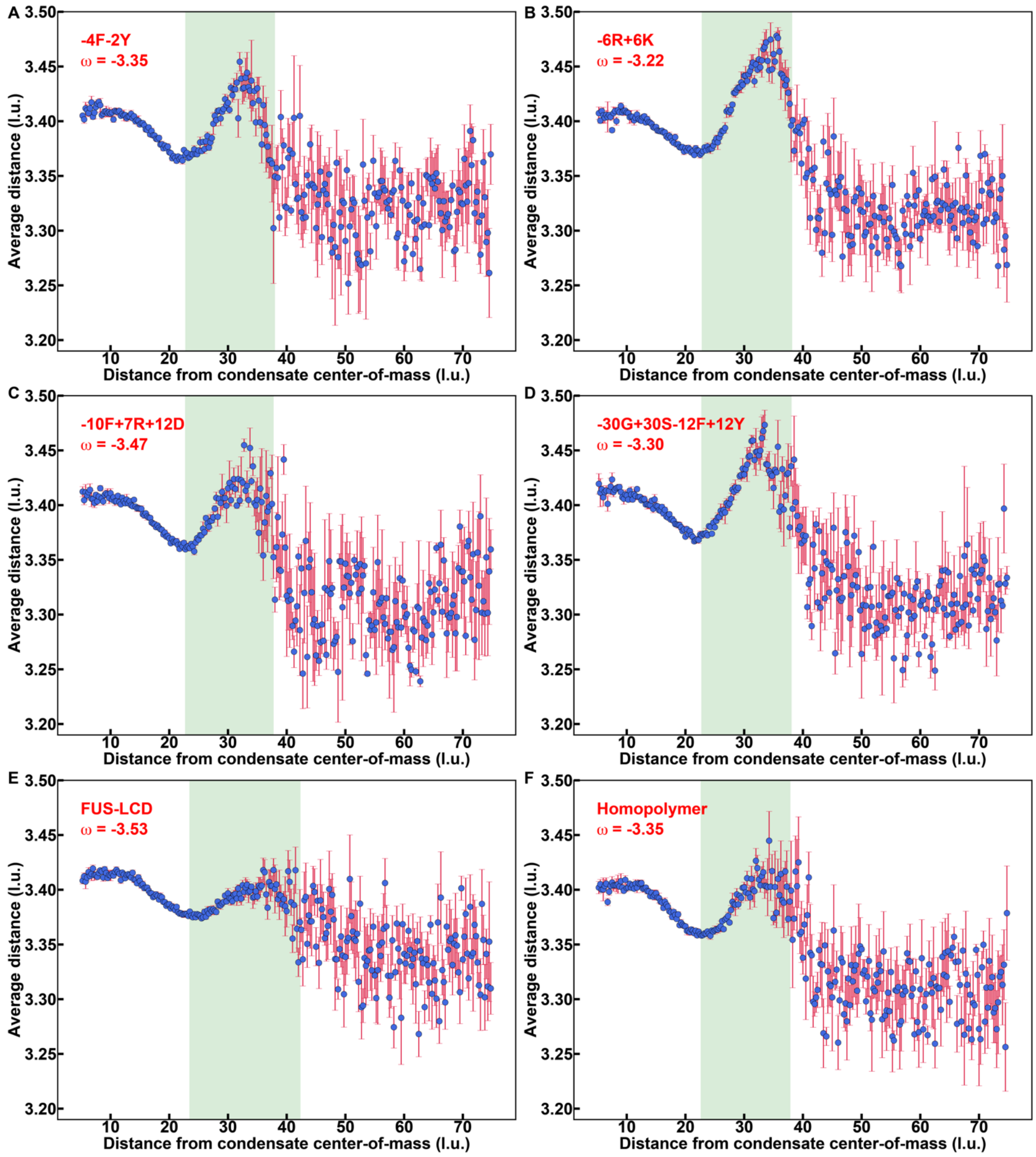

Supplementary Figure 16: Average distance between residues on the same chain that are separated by exactly five residues plotted against the distance from the condensate center-of-mass of one of the residues for various protein constructs. **(A)** -4F-2Y at ω = -3.35, **(B)** -6R+6K at ω = -3.22, **(C)** -10F+7R+12D at ω = -3.47, **(D)** -30G+30S-12F+12Y at ω = -3.22, **(E)** -FUS-LCD at ω = -3.22, **(F)** homopolymer at ω = -3.22. Representative simulations were chosen such that the value of ω was as close as possible to -3.38, the ω value of the WT analyses shown in Fig. 4-5. The translucent green boxes represent the interfacial region as determined by the logistic fit. In all panels, error bars signify standard errors about the mean across 3 replicates and l.u. is lattice units. Source data are provided as a Source Data file.

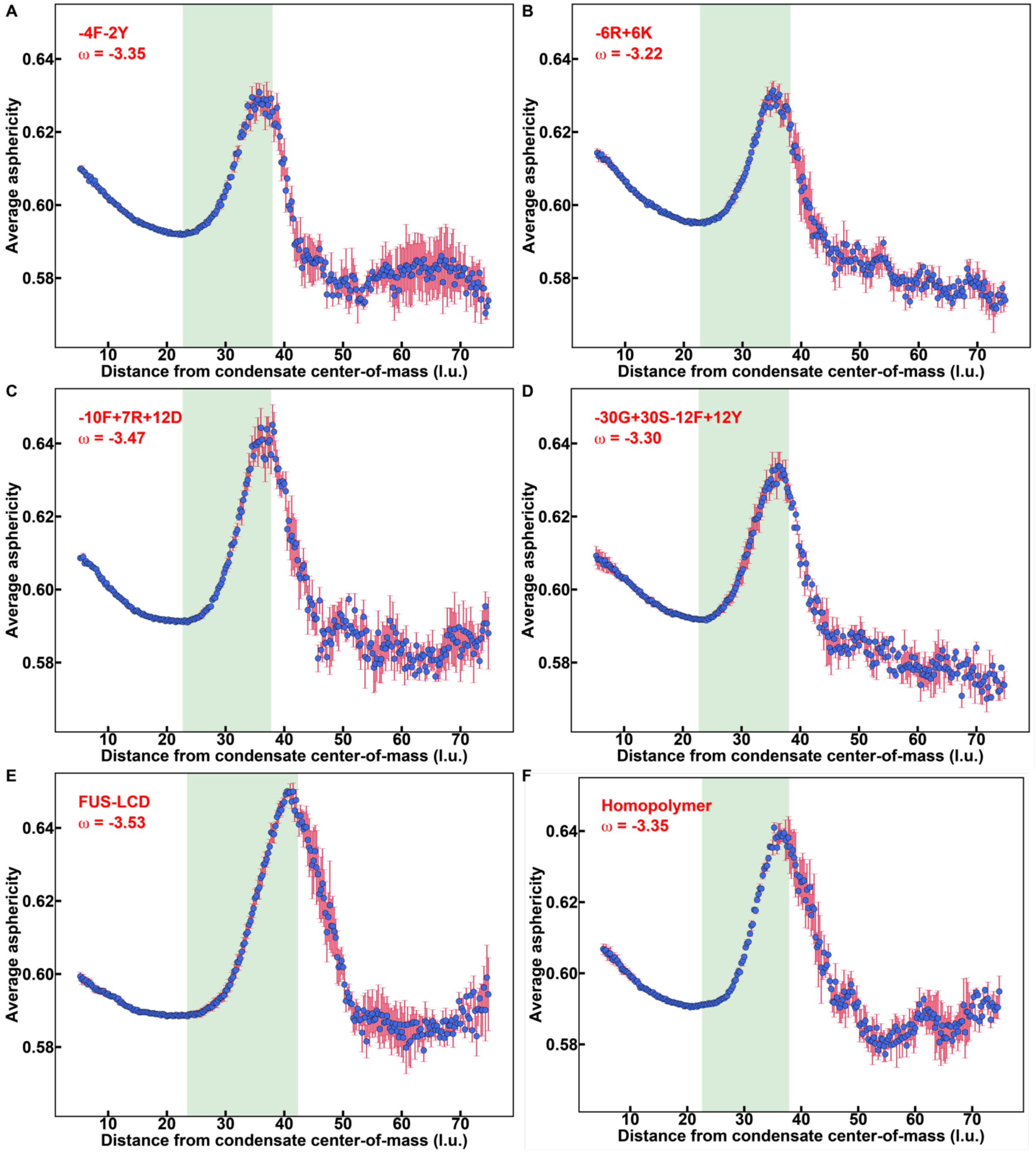

Supplementary Figure 17: Average asphericity of chains plotted against the distance from the condensate center-of-mass for various protein constructs. (A) -4F-2Y at ω = -3.35, **(B)** -6R+6K at ω = -3.22, **(C)** -10F+7R+12D at ω = -3.47, **(D)** -30G+30S-12F+12Y at ω = -3.22, **(E)** -FUS-LCD at ω = -3.22, **(F)** homopolymer at ω = -3.22. Representative simulations were chosen such that the value of ω was as close as possible to -3.38, the ω value of the WT analyses shown in Fig. 4-5. The translucent green boxes represent the interfacial region as determined by the logistic fit. In all panels, error bars signify standard errors about the mean across 3 replicates and l.u. is lattice units. Source data are provided as a Source Data file.

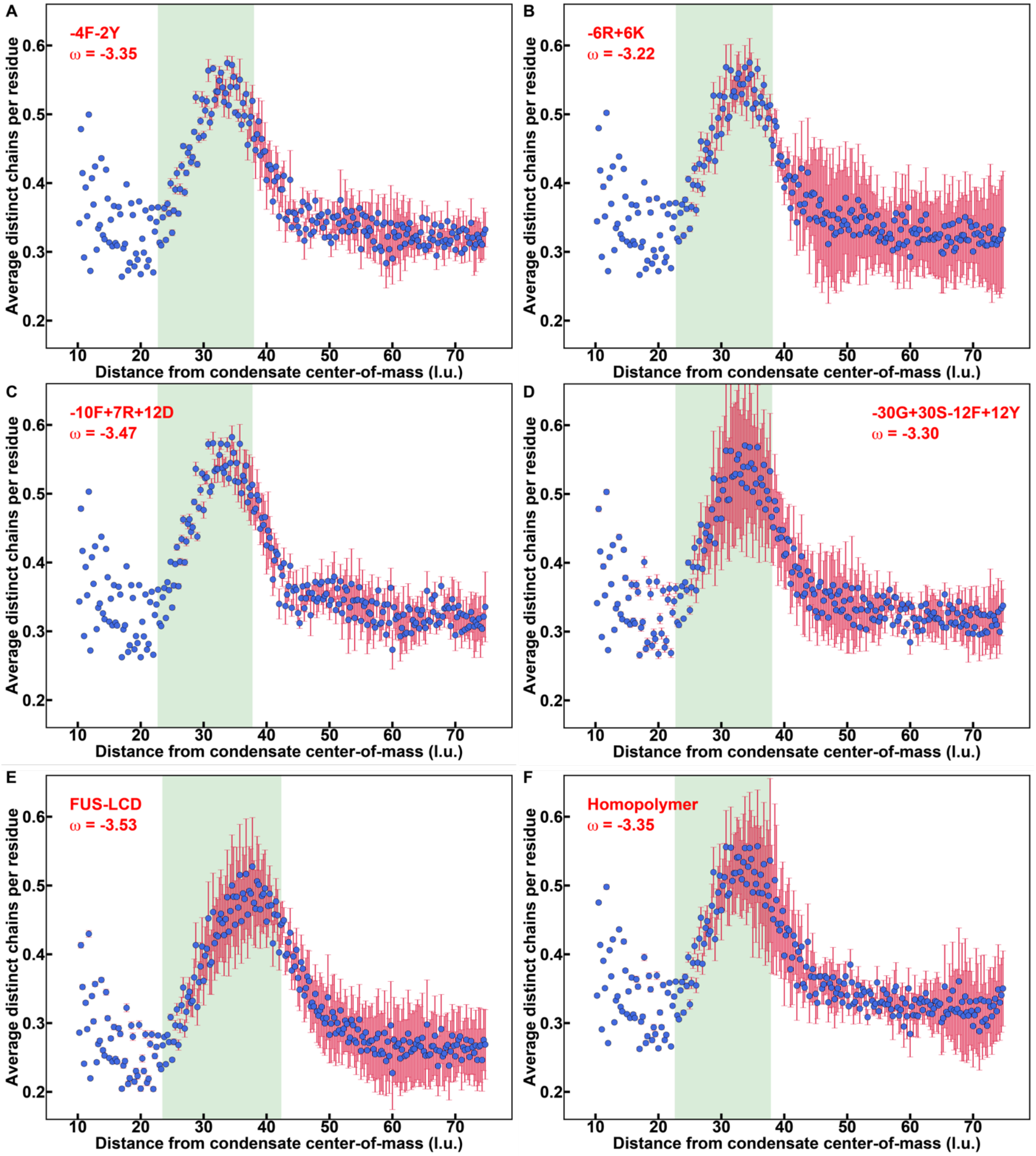

Supplementary Figure 18: Average distinct chains per residue plotted against the distance from the condensate center-of-mass. **(A)** -4F-2Y at ω = -3.35, **(B)** -6R+6K at ω = -3.22, **(C)** -10F+7R+12D at ω = -3.47, **(D)** -30G+30S-12F+12Y at ω = -3.22, **(E)** -FUS-LCD at ω = -3.22, **(F)** homopolymer at ω = -3.22. Representative simulations were chosen such that the value of ω was as close as possible to -3.38, the ω value of the WT analyses shown in Fig. 4-5. The translucent green boxes represent the interfacial region as determined by the logistic fit. In all panels, error bars signify standard errors about the mean across 3 replicates and l.u. is lattice units. Source data are provided as a Source Data file.

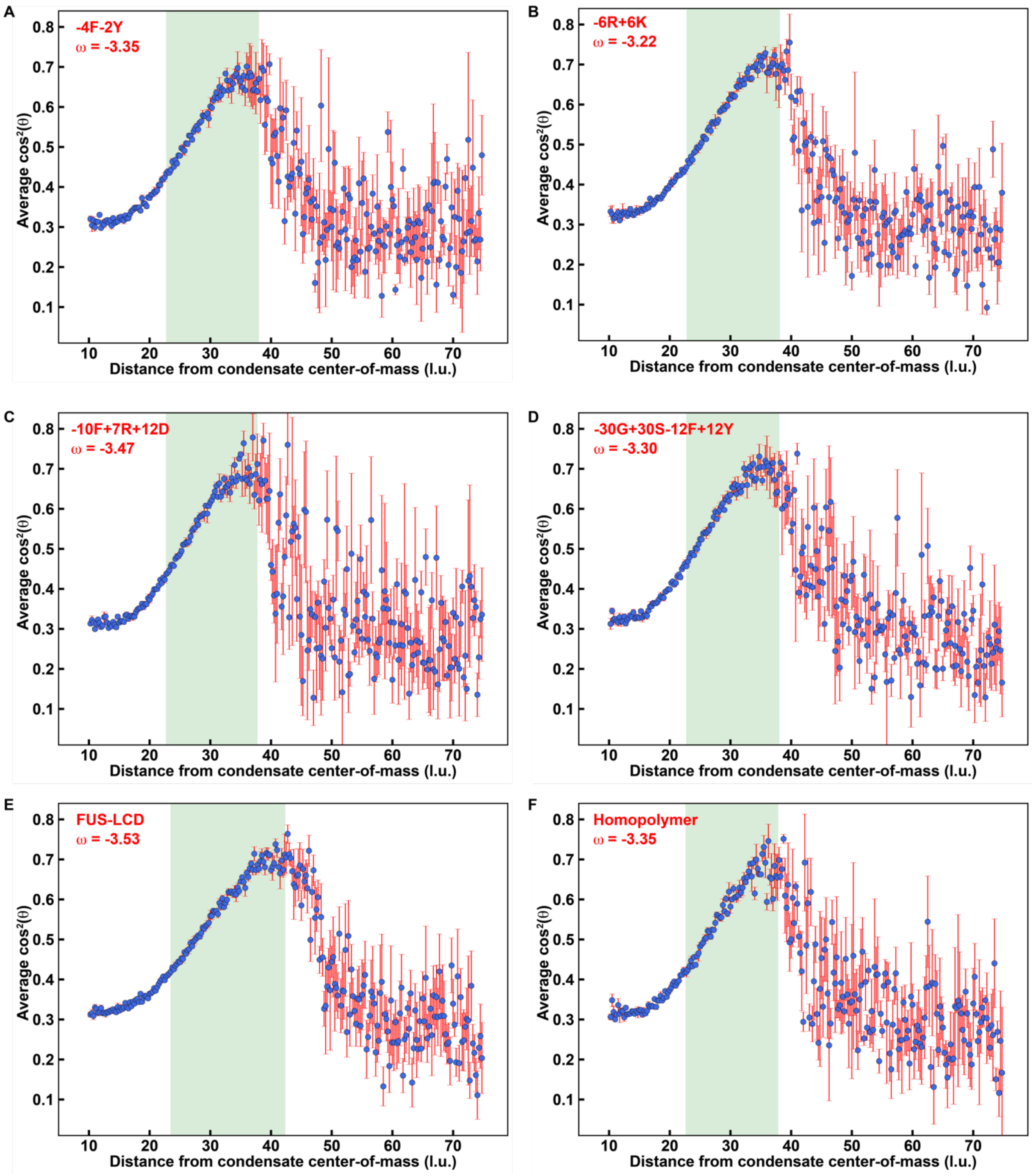

Supplementary Figure 19: Average $\cos^2\theta$ plotted against the distance from the condensate center-of-mass for various protein constructs. **(A)** -4F-2Y at ω = -3.35, **(B)** -6R+6K at ω = -3.22, **(C)** -10F+7R+12D at ω = -3.47, **(D)** -30G+30S-12F+12Y at ω = -3.22, **(E)** -FUS-LCD at ω = -3.22, **(F)** homopolymer at ω = -3.22. Representative simulations were chosen such that the value of ω was as close as possible to -3.38, the ω value of the WT analyses shown in Fig. 4-5. The translucent green boxes represent the interfacial region as determined by the logistic fit. In all panels, error bars signify standard errors about the mean across 3 replicates and l.u. is lattice units. Source data are provided as a Source Data file.

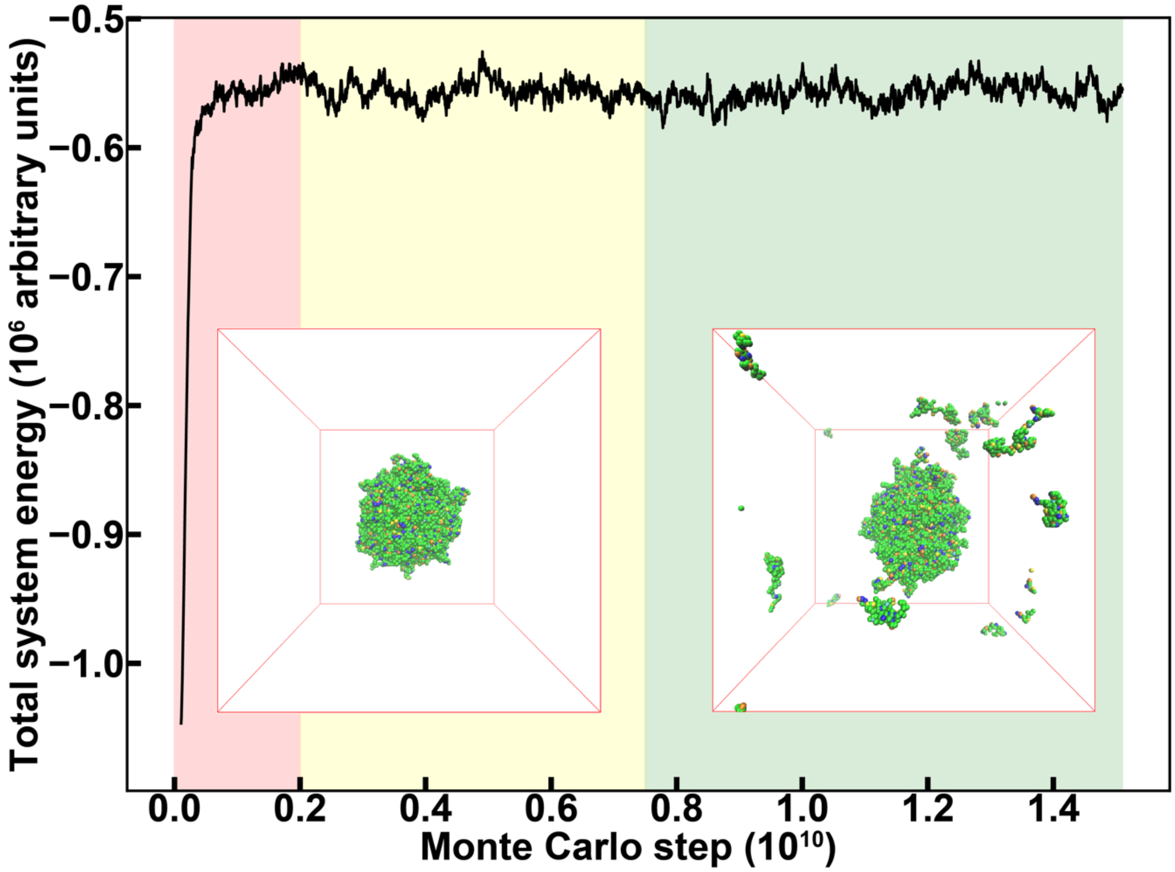

Supplementary Figure 20: Sample energy equilibration of a multi-chain LaSSI simulation. The region in red shows the energy as the system goes from the highly concentrated initialized state to a single equilibrated condensate. The regions in yellow and green show the energy of the equilibrated system. The region in green corresponds to the Monte Carlo steps during which the analyses in this work are performed. Two representative images of LaSSI simulations are also shown. The image on the left shows a condensate in the red zone. The image on the right shows a condensate in the green zone. Source data are provided as a Source Data file.

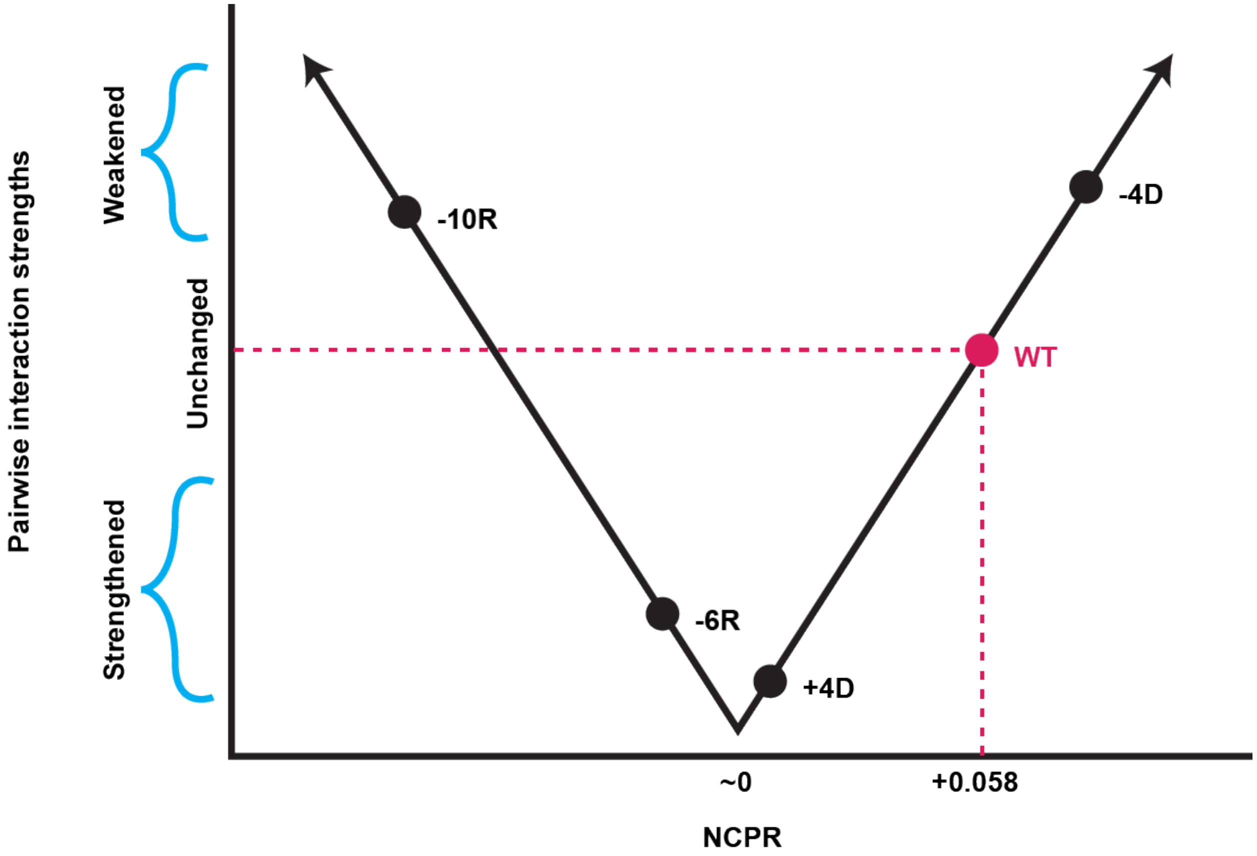

Supplementary Figure 21: A diagram depicting how charge is incorporated in multi-chain simulations. In accordance with previous findings, a high magnitude of net charge per residue (NCPR) will cause a protein to be more soluble and phase separate at a higher concentration. In contrast, a low magnitude of NCPR will cause a protein to be less soluble and phase separate at a lower concentration. We take this into account by comparing the NCPR of a given variant with that of wild-type A1-LCD. As shown in this plot, the NCPR of wild-type A1-LCD is 0.058. If a variant has a significantly higher |NCPR| than 0.058, we weaken the pairwise interaction strengths among protein molecules. Similarly, if a protein has a lower |NCPR|, we strengthen the pairwise interaction strengths. Depicted are 4 other A1-LCD variants: -10R and -4D, whose |NCPR| values are higher than that of the wild-type, resulting in weakened pairwise interactions, as well as -6R and +4D, whose |NCPR| values are lower than that of the wild-type, resulting in strengthened pairwise interactions.

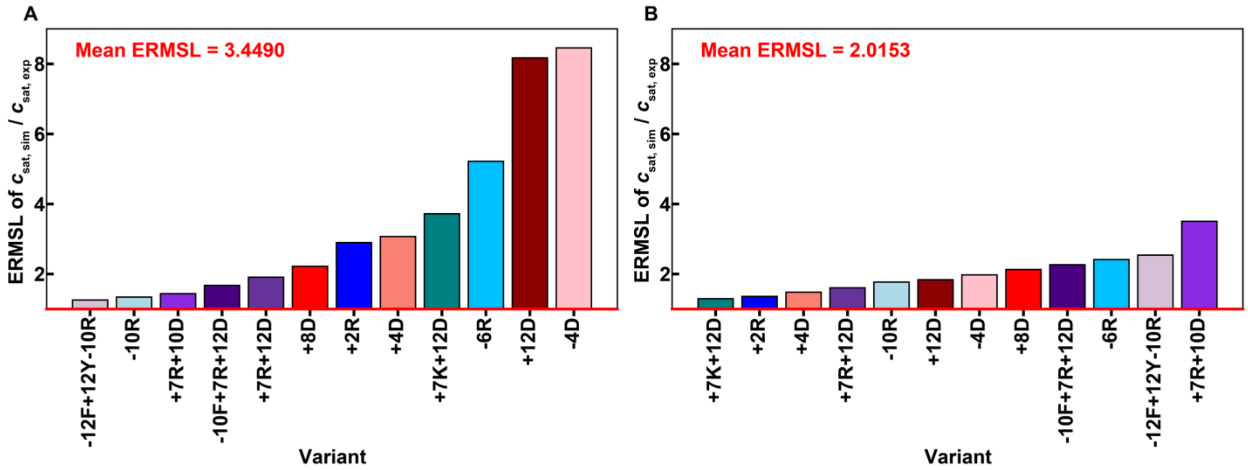

Supplementary Figure 22: ERMSL values to quantify deviations between computed vs. experimentally measured dilute arms of A1-LCD variants without (A) and with (B) a mean-field NCPR-based term. Source data are provided as a Source Data file.

References

1. Choi J-M, Dar F, Pappu RV. LASSI: A lattice model for simulating phase transitions of multivalent proteins. *PLOS Computational Biology* 2019, **15**(10)**:** e1007028.

2. Harmon TS, Holehouse AS, Rosen MK, Pappu RV. Intrinsically disordered linkers determine the interplay between phase separation and gelation in multivalent proteins. *eLife* 2017, **6:** 30294.

3. Bremer A, Farag M, Borcherds WM, Peran I, Martin EW, Pappu RV*, et al.* Deciphering how naturally occurring sequence features impact the phase behaviours of disordered prion-like domains. *Nature Chemistry* 2022, **14:** 196-207.

4. Martin EW, Holehouse AS, Peran I, Farag M, Incicco JJ, Bremer A*, et al.* Valence and patterning of aromatic residues determine the phase behavior of prion-like domains. *Science* 2020, **367**(6478)**:** 694-699.

5. Wei MT, Elbaum-Garfinkle S, Holehouse AS, Chen CC, Feric M, Arnold CB*, et al.* Phase behaviour of disordered proteins underlying low density and high permeability of liquid organelles. *Nature Chemistry* 2017, **9**(11)**:** 1118-1125.

6. Meng W, Lyle N, Luan B, Raleigh DP, Pappu RV. Experiments and simulations show how long-range contacts can form in expanded unfolded proteins with negligible secondary structure. *Proceedings of the National Academy of Sciences USA* 2013, **110**(6)**:** 2123-2128.

7. Ruff K, M., Harmon TS, Pappu RV. CAMELOT: A machine learning approach for coarse-grained simulations of aggregation of block-copolymeric protein sequences. *The Journal of Chemical Physics* 2015, **143**(24)**:** 243123.

8. Pedregosa F, Varoquax G, Gramfort A, Michell V, Thirion B. Scitkit-learn: Machine Learning in Python. *Journal of Machine Learning Research* 2011, **12:** 2825-2830.
